# Multi-omics profiling reveals microRNA-mediated insulin signaling networks

**DOI:** 10.1186/s12859-020-03678-0

**Published:** 2020-09-17

**Authors:** Yang-Chi-Dung Lin, Hsi-Yuan Huang, Sirjana Shrestha, Chih-Hung Chou, Yen-Hua Chen, Chi-Ru Chen, Hsiao-Chin Hong, Jing Li, Yi-An Chang, Men-Yee Chiew, Ya-Rong Huang, Siang-Jyun Tu, Ting-Hsuan Sun, Shun-Long Weng, Ching-Ping Tseng, Hsien-Da Huang

**Affiliations:** 1 School of Life and Health Sciences, The Chinese University of Hong Kong, Longgang District, Shenzhen, 518172 Guangdong Province China; 2 Warshel Institute for Computational Biology, The Chinese University of Hong Kong, Longgang District, Shenzhen, 518172 Guangdong Province China; 3 Institute of Bioinformatics and Systems Biology, National Chiao Tung University, Hsinchu, 300 Taiwan; 4 Department of Biological Science and Technology, National Chiao Tung University, Hsinchu, 300 Taiwan; 5 Department of Microbiology and Immunology, Weill Cornell Medicine, Cornell University, New York, NY 10021 USA; 6 Department of Obstetrics and Gynecology, Hsinchu Mackay Memorial Hospital, Hsinchu, 300 Taiwan

**Keywords:** miRNA, Pancreatic beta-cell, RNA-seq, Multi-omics

## Abstract

**Background:**

MicroRNAs (miRNAs) play a key role in mediating the action of insulin on cell growth and the development of diabetes. However, few studies have been conducted to provide a comprehensive overview of the miRNA-mediated signaling network in response to glucose in pancreatic beta cells. In our study, we established a computational framework integrating multi-omics profiles analyses, including RNA sequencing (RNA-seq) and small RNA sequencing (sRNA-seq) data analysis, inverse expression pattern analysis, public data integration, and miRNA targets prediction to illustrate the miRNA-mediated regulatory network at different glucose concentrations in INS-1 pancreatic beta cells (INS-1), which display important characteristics of the pancreatic beta cells.

**Results:**

We applied our computational framework to the expression profiles of miRNA/mRNA of INS-1, at different glucose concentrations. A total of 1437 differentially expressed genes (DEGs) and 153 differentially expressed miRNAs (DEmiRs) were identified from multi-omics profiles. In particular, 121 DEmiRs putatively regulated a total of 237 DEGs involved in glucose metabolism, fatty acid oxidation, ion channels, exocytosis, homeostasis, and insulin gene regulation. Moreover, Argonaute 2 immunoprecipitation sequencing, qRT-PCR, and luciferase assay identified Crem, Fn1, and Stc1 are direct targets of miR-146b and elucidated that miR-146b acted as a potential regulator and promising target to understand the insulin signaling network.

**Conclusions:**

In this study, the integration of experimentally verified data with system biology framework extracts the miRNA network for exploring potential insulin-associated miRNA and their target genes. The findings offer a potentially significant effect on the understanding of miRNA-mediated insulin signaling network in the development and progression of pancreatic diabetes.

## Background

MicroRNAs (miRNAs) are short, noncoding RNAs of approximately 22 nucleotides in length that function as important regulators at the post-transcriptional level either by degrading mRNA molecules or suppressing protein translation [1]. miRNAs play critical roles in regulating numerous physiological processes, including the cell cycle [2], cell growth, development, differentiation [3], apoptosis [4], and pathological processes, such as those associated with various cancers [5]. Recent advances in multi-omics profiling technologies have fundamentally changed the process of large-scale screening in miRNA studies. Different techniques, such as RNA sequencing (RNA-seq), small RNA sequencing (sRNA-seq), Argonaute immunoprecipitation sequencing (Ago IP-seq), Argonaute cross-linking and immunoprecipitation sequencing (CLIP-seq) and chromatin immunoprecipitation sequencing (ChIP-seq) can facilitate discovery of novel miRNAs, miRNA–target interactions (MTIs), and miRNA regulation by transcription factors (TF). In addition, miRTarBase 7.0 [6] houses 4076 mature miRNAs and about 422,517 MTIs that are supported by considerable strong experimental evidence.

Diabetes is a metabolic disorder occurring in patients with high blood glucose level or hyperglycemia, which brings serious complications, such as heart and kidney diseases. Pancreatic beta - cells are responsible for the secretion of insulin by which the blood glucose level is controlled [7]. High glucose infusion in rats can lead to an approximately 40–60% increment in mass and proliferation of beta-cells [8]. Several intracellular signaling pathways, such as the phosphatidylinositol 3-kinase (PI3K)/Akt pathway [9], can induce beta-cell proliferation and is correlated with blood sugar. Recently many studies have identified several miRNAs as pathological factors, which contribute to the development of diabetes [10–12]. For example, miR-375 is an abundantly found miRNA in beta-cells [13] and increased expression of this miRNA is observed in patients with type 2 diabetes (T2D) [14]. Moreover, overexpression of miR-375 down-regulates 3phosphoinositide-dependent protein kinase-1 [15] and decreases glucose-stimulated insulin secretion whereas its up-regulation is correlated with beta-cell mass loss [16]. Substantial accumulating evidence indicates that miRNAs can perform regulatory roles of cell proliferation and diabetes through glucose-stimulated response in pancreatic beta-cells [17, 18]. The secretion of insulin is negatively regulated by miR-375 and the repression of miR-375 through the cAMP-PKA pathway may enhance the insulin response in pancreatic beta-cells [19]. Due to repression in miR-7, mTOR-signaling gets activated, which in turn promotes beta-cell proliferation in both human and mouse [20]. However, miR-7 functions in supporting the survival of beta-cell at the embryonic developmental stage. The delivery of miR-7 morpholinos to early mouse embryos results in a decrease of the total beta-cells number and their dysfunction [21]. miR-124a-2, another important regulator of embryonic pancreatic development, targets Foxa2, the activator of pancreatic duodenal homeobox 1 involved in the differentiation and survival of pancreatic beta cells [22]. Defected miR-124a-2 may be associated with beta-cell dysfunction [23]. Even though numerous studies have provided evidence that glucose- stimulated insulin secretion (GSIS) can be mediated by miRNAs, the comprehensive molecular mechanisms of miRNA-mediated gene regulation in glucose-stimulated pancreatic beta cells remain poorly understood. MTIs analysis has become a widely adopted approach to unravel target genes and pathways in biological systems.

Recently several web servers and online bioinformatics tools have been developed for the study of MTIs and miRNA regulatory networks. For example, dChip-GemiNi [24] is a web server that can be used to identify miRNA, TF, and their target gene networks with gene and miRNA expression profiles. Another web server, mirConnX [25] provides mRNA and miRNA gene regulatory networks through the computational prediction of MTIs and TF-miRNA. Similarly, MAGIA [26] provides platforms to construct TF-miRNA regulatory networks. All of these tools offer an integrated gene and miRNA expression profiles with target prediction. For confirming the uncertainty of miRNA target prediction tools, high-throughput experimental techniques for MTIs with Ago IP-seq and TF-miRNA association studies with ChIP-seq are widely applied in the study of miRNA-gene regulatory networks [27, 28].

To facilitate the study of novel MTIs in glucose-stimulated beta cells and to discover new insights into diabetes, we used an integrative multi-omics and systems biology approach, including RNA sequencing (RNA-seq) and small RNA sequencing (sRNA-seq) at different glucose concentrations to reconstruct the miRNA-mediated gene regulatory network in INS-1 pancreatic beta cells (INS-1). INS-1 cells display important characteristics of the pancreatic beta cells, including insulin expression, stable pancreatic beta-cell phenotype over 116 passages, and responsiveness to glucose within the physiological range [29]. Due to the above reasons, we selected INS-1 as the model cell line to investigate the MTIs in this study.

In this study, we combined the multi-omics profiles, public MTI database, TF-miRNA database, and computational prediction tools to enhance the identification of MTIs. Based on our newly constructed miRNA-gene regulatory network, we further performed the large-scale MTIs validation based on Ago2 IP-seq methodology [30] to rule out the possibility of false-positive MTIs. In addition, we further validated that miR-146b which inhibited by high glucose concentration can promote the regulation of insulin release-related genes. In brief, this study reveals a novel MTIs in INS-1 under increasing glucose conditions and clarifies the molecular mechanisms associated with insulin signaling network in the pancreatic beta-cell.

## Results

**Fig. 1:**
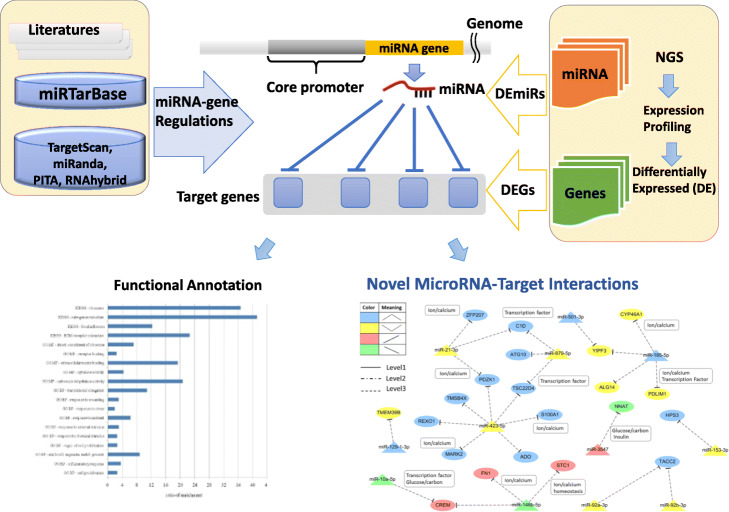
Schematic overview of the computational approaches to reconstruct the miRNA-gene regulatory network

Figure 1 presents an overview of the proposed computational framework to identify the novel MTIs in INS-1 under different glucose concentrations by high-throughput expression profiles. The method includes the following two steps: (1) identification of differentially expressed genes (DEGs) and differentially expressed miRNAs (DEmiRs) from RNA-seq and small RNA-seq data, respectively; (2) mapping of DEGs and DEmiRs to the curated novel MTIs and reconstruction of the regulatory network and functional annotation of the target genes.

### Effects of glucose concentration on INS-1 growth

INS-1 doubling time was between 50 and 100 h depending on the glucose concentration of the culture medium [31]. The speed of cell division was affected by glucose levels, and the average subculture time was 1 week. Insulin secretion and complete cell morphology were retained even after 80 generations of the successive subculture; thus, the cells were considered stable for the study. Subculture generations 4 to 10 were used in the study. Cell morphology and growth pattern were observed under a microscope (Figure S1). A high glucose level of 30 mM was beneficial to INS-1 adhesion, but a massive suspension of INS-1 was observed on day 4 under a low glucose level of 2 mM (Figure S1). To obtain a detailed understanding of the effect of glucose on INS-1 growth, we measured the cell growth curve and doubling time. The INS-1 doubling times were 102.76, 51.63, and 45.69 h at 2 mM, 11.1 mM, and 30 mM glucose concentration, respectively (Figure S2). The cell doubling time under moderate or high glucose level was found to be consistent with the values in the literature [31].

### Effect of glucose concentration on miRNAs and genes expression in INS-1

**Fig. 2:**
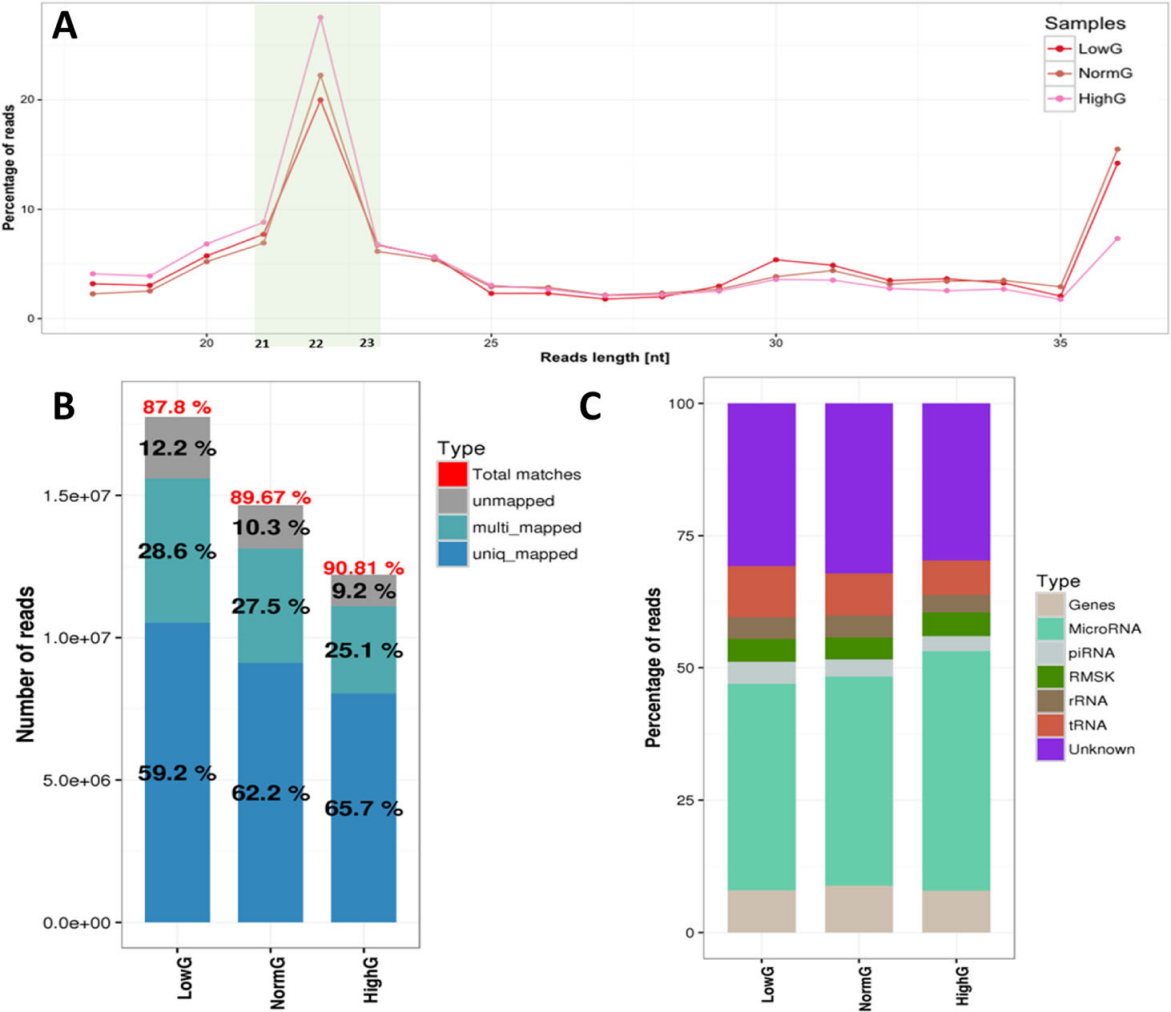
Read quality and distribution of small RNA-seq data. **a** The read length distribution shows that all samples reached a peak miRNA length **b** Mapping ratio of reads to the reference genome **c** Propor-tion of reads (> 40%) detected from miRNAs

sRNA-seq and RNA-Seq are highly sensitive and accurate tools for measuring miRNAs and mRNAs expression across the transcriptome under different environmental conditions. In our study, there were three RNA samples under three increasing glucose conditions (2 mM, low glucose; 11.1 mM, moderate glucose; and 30 mM, high glucose) for sRNA and mRNA sequencing to observe the DEGs and DEmiRs. The read quality of the sRNA-seq and RNA-seq are shown in Fig. 2 and Figure S3. The read length distribution line chart indicates that all samples reached a peak miRNA length in Fig. 2a. More than 87% reads were mapped to the reference genome in Fig. 2b. Figure 2c displays more than 40% of reads were obtained from miRNAs. On the other hand, the read quality of RNA-seq present in Figure S3A and more than 91% reads were mapped to the reference genome. Figure S3B present the DEGs analyzed from RNA-seq data using Volcano plot. The detailed information about differentially expressed miRNAs and mRNAs are provided in Additional file 1 and Additional file 2. Furthermore, miRNAs were identified as significantly and differentially expressed relative to those at moderate glucose concentration if the fold change exceeded or was equal to 1.4 (upregulated miRNAs) or was less than or equal to 0.71 (downregulated miRNAs). mRNAs were identified as significantly and differentially expressed relative to moderate glucose concentration if the fold change ≥ 1.5 (upregulated mRNAs) or ≤ 0.67 (downregulated mRNAs).

**Fig. 3:**
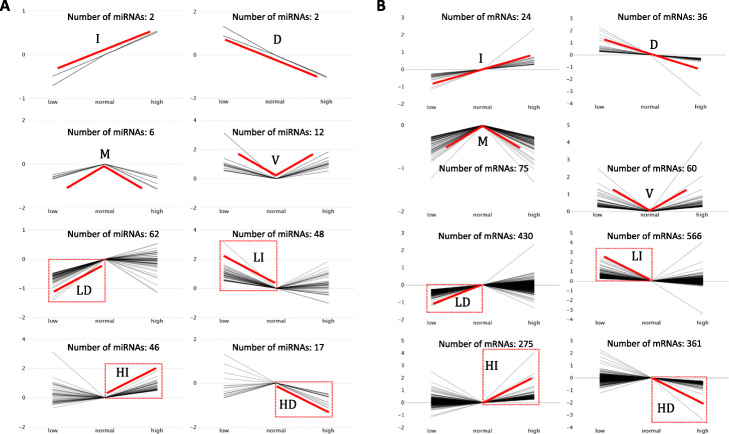
Eight types of (**a**) miRNA and (**b**) mRNA gene expression profiles in response to different glucose concentrations. The x-axis shows three different glucose concentrations (low, normal, and high). The y-axis shows the log2 fold change compared with normal glucose concentration. The red line represents the trends of the expression. I (increased continuously); D (decreased continuously); M (decreased under both low and high glucose conditions); V (increased under both low and high glucose conditions); LD (decreased under low glucose and no significant expression change under high glucose conditions); LI (increased under low glucose and no significant expression change under high glucose conditions); HI (increased under high glucose conditions and no significant expression change under low glucose); HD (decreased under high glucose conditions and no significant expression change under low glucose)

To analyze the experimental results of the miRNA expression and their target genes, we defined eight types of expression profiles according to their responses to different glucose concentrations. Figure 3 demonstrates different profile categories for miRNA (Fig. 3a**)** and mRNA (Fig. 3b**)** expression. The gene expression profiles of low and high glucose conditions were correlated with moderate glucose concentration. The eight types of expression profiles were named as follows: **I** (increased continuously); D (decreased continuously); M (decreased under both low and high glucose conditions); V (increased under both low and high glucose conditions); LD (decreased under low glucose and no significant expression change under high glucose conditions); LI (increased under low glucose and no significant expression change under high glucose conditions); HI (increased under high glucose conditions and no significant expression change under low glucose); HD (decreased under high glucose conditions and no significant expression change under low glucose).

Under our assumption that most miRNAs act as a negative regulator of their target mRNAs, each type of miRNA and its target should present an inverse expression profile. For example, the inverse expression of the I type was the D type and that of the HD type was the HI type. The number of genes and miRNAs in each type are shown in Fig. 3a and b, respectively. To obtain high-confidence MTI pairs from the high-throughput data, we defined MTIs by using experimental or predicted evidence and miRNA and its target gene with the inverse expression level. Eight types of MTI pairs with high confidence were observed and further analyzed to construct the network and function.

In this study, RNA and small RNA sequencing techniques were applied to obtain DEGs and DEmiRs for each glucose concentration in which INS-1 cells were incubated. To validate the expression profiling of DEGs and DEmiRs, we selected nine genes (Glp1r, Calr, Pde4b, Cited2, Trpc4, Crem, Ins1, Rasgrp2, and Camk) and two miRNAs (miR-375 and miR-16) on the basis of the reports published in the literature [32]. Then, quantitative reverse-transcription PCR (qRT-PCR) was conducted to verify the expression profile of these genes in RNA samples used in next-generation sequencing and another three independent RNA samples under three culture conditions at different glucose concentrations. The results of qRT-PCR validation (Figure S4A) show that the experimental design is highly relevant, and the trend of differential gene expression induced by glucose is consistent with that of the RNA-seq profiles FPKM (fragments per kilobase of transcript per million mapped reads) value (Figure S4B).

### Novel MTIs analysis of miRNA-mediated gene regulatory in different glucose concentration stimulated

**Fig. 4:**
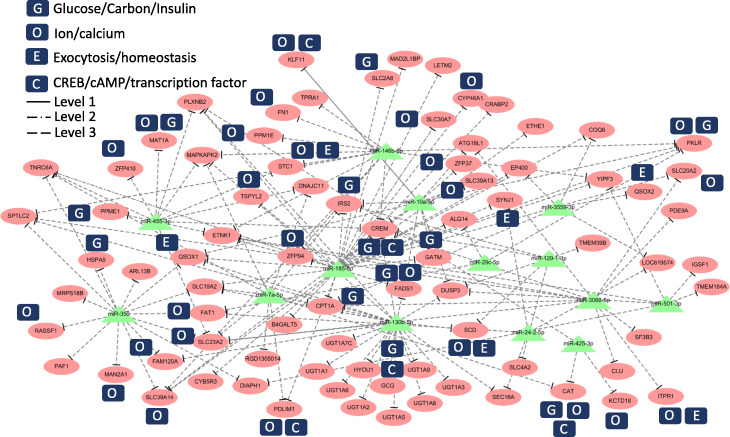
DN-miRNA-mediated gene regulatory network in high glucose concentration. Level 1 represents the interactions of miRNA targets with strong experimental evidence (supported by luciferase reporter assay or Western blot). Level 2 represents the interactions of miRNA targets with substantial weaker experimental evidence (supported by CLIP-seq experiments). Level 3 represents the interactions of the predicted miRNA targets. The oval denotes mRNA, the triangle represents miRNA

**Fig. 5:**
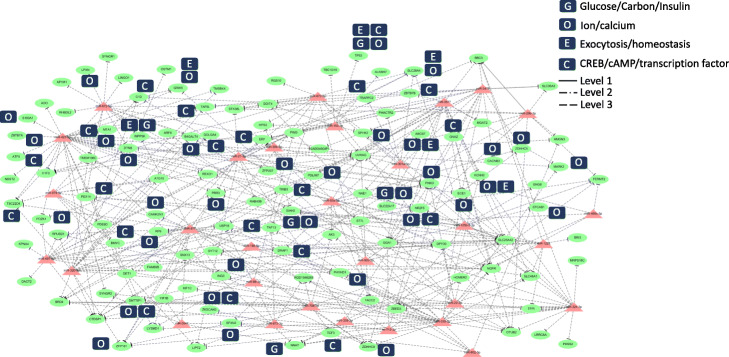
UP-miRNA-mediated gene regulatory network at high glucose concentration. Level 1 represents the interactions of miRNA targets with strong experimental evidence (supported by luciferase reporter assay or Western blot). Level 2 represents the interactions of miRNA targets with substantial weaker experimental evidence (supported by CLIP-seq experiments). Level 3 represents the interactions of the predicted miRNA targets. The oval denotes mRNA, the triangle represents miRNA

On the basis of the previous report [33], we selected genes with the following keywords: glucose/carbon/insulin (indicated as G), ion/calcium (indicated as O), exocytosis/homeostasis (indicated as E), and CREB/cAMP/transcription factor (indicated as C) [34–39]; and then, we conducted network analysis to find the novel miRNA-mediated gene regulatory network under glucose variation in Figs. 4, 5, S5 and S6. In these figures, the solid line (Level 1) represents the interactions of miRNA targets with strong experimental evidence (supported by luciferase reporter assay or Western blot); dotted/dashed line (Level 2) represents the interactions of miRNA targets with substantial weaker experimental evidence (supported by CLIP-seq experiments); dashed line (Level 3) represents the interactions of the predicted miRNA targets.

The downregulated miRNA (DN-miRNA)-mediated gene regulatory network in high glucose involved 14 DN-miRNAs and 80 upregulated genes, which were associated with the selected gene function (G, O, E, and C) (Fig. 4). The results of our study indicated that miR-185-5p and miR-10a-5p were the key miRNAs that regulated more than 10 genes under high glucose conditions. The upregulated miRNA (UP-miRNA)-mediated gene regulatory network in high glucose involved 29 UP-miRNAs and 51 downregulated genes, which were associated with the selected gene function (G, O, E, and C) (Fig. 5). miR-423-5p, miR-3577, miR-21-3p, and miR-320-3p were the key regulators that suppressed more than 10 genes. The differentially expressed miRNA (DE-miRNA)-mediated gene regulatory network at low glucose concentration are shown in Figures S5 and S6. We used gene functional annotation and analyzed the network under different conditions because of the large number of genes involved in different types of networks.

We summarized the top 20 gene ontology (GO) results of different glucose-stimulated types of the miRNA-mediated gene regulatory network. We found that DN-miRNA-mediated genes under high glucose conditions (Figure S7) were associated with the organelle membrane (*p*-value: 4.01E− 4), response to starvation (*p*-value: 0.001) and nutrient levels (p-value: 0.002) under biological process categories. UP-miRNA-mediated genes under high glucose conditions (Figure S8) were majorly associated with the transcription regulator activity (*p*-value: 8.30E− 4) under the categories of biological process and molecular function. Cell cycle-related genes (*p*-value: 1.12E− 10) were downregulated at low glucose levels (DN-miRNA-mediated genes in low glucose concentration, Figure S9); this result is in accordance with the slow growth rate of INS-1 cells when cultured under low glucose condition. The genes were induced by glucose starvation (UP-miRNA-mediated genes in low glucose concentration Figure S10) via the endoplasmic reticulum-unfolded protein response, which protects cells from cell death (*p*-value: 2.90 E × 10^5^).

**Fig. 6:**
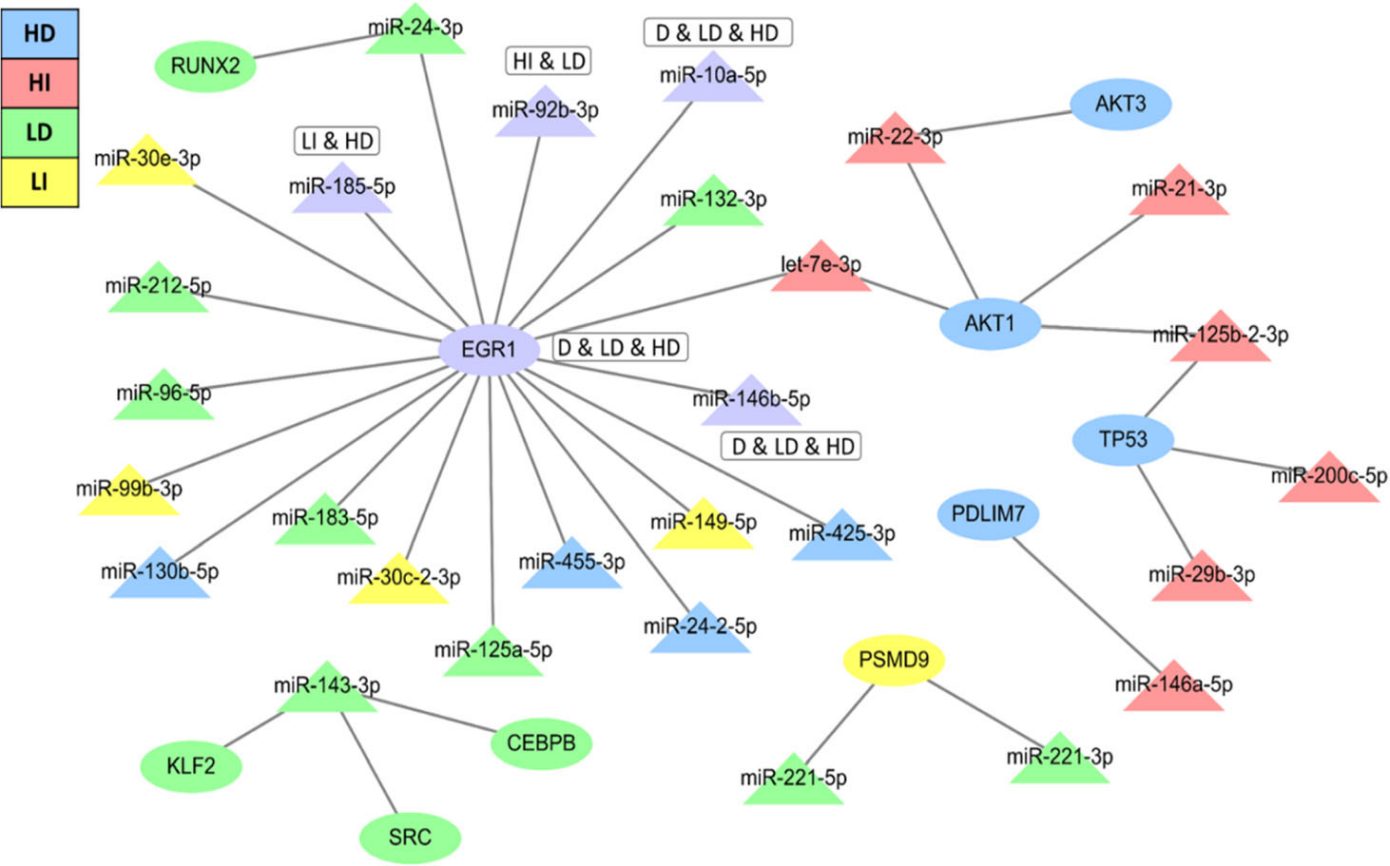
TF-miRNA regulatory networks in pancreatic beta cells. (HD- decreased under high glucose condition, HI-increased under high glucose condition, LD-decreased under low glucose condition, LI-increased under low glucose condition). The oval denotes mRNA, the triangle represents miRNA

In addition, we established TF-miRNA regulatory networks in pancreatic beta cells. Thirty-four TF-miRNA interactions, including 10 TFs and 28 DE-miRNAs, were identified using TransmiR and gene/miRNA expression profiling Fig. 6 and these results indicated EGR1 as a key transcription factor regulating 19 DE-miRNAs.

### High-throughput identification of miRNA-target interactions by Ago2 Immunoprecipitation (Ago2 IP)

**Fig. 7:**
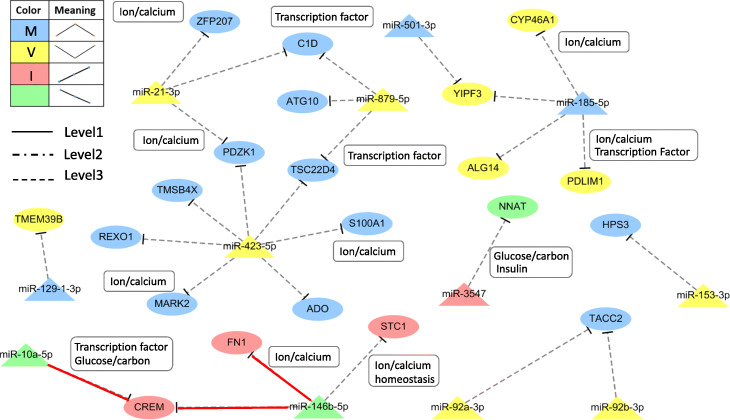
DE-miRNA-mediated gene regulatory network under glucose stimulation. The dashed (Level 3) represents the interactions of the predicted miRNA targets under glucose stimulation and the solid red line (Level 4) indicated the MTIs which validated by Ago2 IP-seq experiment. The oval denotes mRNA, the triangle represents miRNA

In silico prediction of miRNA targets is an extremely useful approach to identify potential mRNA targets from sequencing data, however, the large number of MTIs still need further experimental validation. The identification of miRNA targets is experimentally laborious and time-consuming. Recently, high-throughput sequencing methods are combined with Ago2 IP-seq to identify miRNAs associated with Ago2. In order to reduce the number of possibilities of MTIs, we further validated the MTIs using Ago2 IP-seq under high or low glucose conditions. Combining the Ago2 IP-seq results, we successfully reduced the number of MTIs in INS-1 pancreatic beta-cells under high glucose concentration. The original number of MTIs from Fig. 4 (151 MTIs), Fig. 5 (331 MTIs), Figure S4 (668 MTIs), and Figure S5 (1013 MTIs), has been reduced to 11 (Additional file 3: Table S1), 85 (Additional file 3: Table S2), 197 (Additional file 3: Table S3), and 314 (Additional file 3: Table S4), respectively. In Fig. 7, we summarized the gene analysis results of different glucose-stimulated types of miRNA-mediated gene regulatory network. The dashed (Level 3) represents the interactions of the predicted miRNA targets under glucose stimulation and the solid red line (Level 4) indicated the MTIs which validated by Ago2 IP-seq experiment.

### Regulatory role of miR-146b on insulin-related genes

**Fig. 8:**
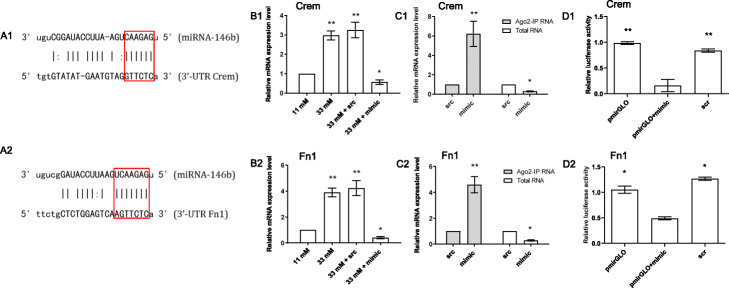
Validation of the two target genes Crem and Fn1 of miR-146b that are common in prediction and RNA-seq analysis. **a** Predicted target sites of miR-146b in 3′-UTR of rat Crem and Fn1. **b** mRNA expression analysis of two target genes of miR-146b by qRT-PCR **c** Ago2-IP analysis was carried out to measure relative enrichment of Crem and Fn1 genes of miR-146b as measured by qRT-PCR. src-scramble. **d** Luciferase activity in INS1 cells cotransfected with either empty luciferase vector pmiR-GLO as positive control or the construct containing 3’UTR of two target genes Crem and Fn1 and miR-146b mimics (mimic) or scramble control (scr)

On the basis of the novel reconstructed miRNA-mediated gene regulatory network (Fig. 7), we further selected and validated two MTIs of Crem and Fn1 to miR-146b by miR-146b mimic transfection, independent Ago2 IP, and qRT-PCR experiments. Notably, the two genes are related to the GSIS mechanism, but no evidence supports that miR-146b can participate in the simultaneous regulation of these genes. The potential target sites of Crem and Fn1 of miR-146b are shown in Fig. 8a. To investigate the regulation of miR-146b target genes, we overexpressed miR-146b and measured the expression levels of Crem and Fn1 using qRT-PCR. The qRT-PCR results confirmed that the mRNA expression of the targets genes was downregulated by miR-146b mimics in comparison with the scramble control (Fig. 8b). Furthermore, as mentioned above, RNA fractions were concentrated using the Ago2 IP method and subjected to miRNA target validation. Either miR-146b mimic or the miR-146b scrambled control (src) was delivered to INS-1 cells with low miR-146b expression level under high glucose condition. Then, qRT-PCR was used to compare the expression levels of Crem and Fn1 mRNAs. The results show that the expression levels of Crem and Fn1 were enriched in the Ago2 IPRNA fractions, whereas low expression of Crem and Fn1 was observed in the total RNA fractions, probably due to the RISC cleavage (Fig. 8c). Moreover, to determine whether miR-146b can repress Crem and Fn1 by targeting its binding site at 3’UTR, we inserted the PCR product containing full length of 3’UTR of two target genes into pmiRGLO luciferase reporter vector. Considering that the translation of target genes are affected by miRNA, we performed dual luciferase reporter assay with INS1 cells co-transfected with either empty luciferase vector or the construct containing 3’UTR of five target genes individually and miR-146b mimics or scramble control. The luciferase assay data indicated that the luciferase reporter activities of Crem and Fn1 were markedly repressed by miR-146b mimics in comparison to scramble control (Fig. 8d). The repression of luciferase reporter assay might be due to binding of miR-146b to the 3’UTR of these target genes. These results suggest that miR-146b might target Crem and Fn1 via their 3’UTR.

## Discussion

Hypoglycemia is a metabolic disorder condition characterized by abnormally low blood glucose levels of usually less than 70 mg/dL. Hypoglycemia is referred to as an insulin reaction or insulin shock. By contrast, hyperglycemia is a medical condition characterized by high blood glucose. When diabetes occurs the blood sugar level fluctuates periodically [40, 41]. Several glucose-dependent genes in pancreatic beta cells have been reported to date. For example, Txnip is a key regulatory factor for apoptosis and diabetic beta-cell dysfunction and prevents T1D and T2D in a rat model. Previous studies showed that Txnip is strongly induced by glucose. In this study, RNA-seq analysis showed that Txnip gene expression increased by 17 times with elevated glucose level from 2 mM to 30 mM. The regulatory mechanism of Txnip shows that transcription regulation initiated by glucose is highly correlated with miRNA [42].

Accumulating data suggest the important role of miRNA in diabetes [43]. We found that the glucose level-associated miRNAs identified using our workflow matched the previously reported diabetes-associated miRNA, such as miR-34, miR-141, and miR-200c [44]. The expression levels of miR-200 family miRNAs, which include miR-141 and miR-200c, were reportedly upregulated in *db/db* mice and induce the upregulation of apoptosis genes. In addition to its function in diabetes, miR-200c facilitates the regulation of the expression of class III beta-tubulin (TUBB3) under the hypoglycemic condition in ovarian cancer [45]. Our present study found that the miR-200 family miRNAs in the INS-1 cell line were downregulated with the increase in environmental glucose concentration, suggesting that the miR-200c family may also play a role in hypoglycemia.

Several miRNAs identified in T2D patients may be useful biomarkers for the disease progression of T2D and treatment response to antidiabetic medications. miR-185-5p is one of the most significantly decreased miRNAs. The effect of decreased miR-185 in diabetes patients remains unclear [46]. In our study, we found that low miR-185-5p expression level at both low and high glucose concentrations. According to the miRNA–mRNA–target interaction analysis, we found four predicted target genes that were regulated by miR-185-5p. Three of the genes (Cyp46a1, Pdlim1, and Alg14) control the synthesis of cholesterol and long-chain fatty acids. The dominant factor in the pathogenesis of T2D is insulin resistance which in turn increases fatty acid and cholesterol of plasma membranes [47].

Moreover, Cyp46a1 encodes a member of the cytochrome P450 superfamily of enzymes. Cytochrome P450 proteins have monooxygenase activity and catalyze a large number of reactions which are involved in drug metabolism as well as the synthesis of cholesterol, steroids, and other lipids [48]. p75 neurotrophin receptor (p75NTR/CD271) mediates glioma invasion that requires regulated interaction with PDLIM1 [49]. Similar to other tumors, gliomas are believed to primarily metabolize glucose for energy production. However, the dependence of gliomas on glycolysis has recently gained attention because fatty acid oxidation enzymes are present and active within glioma tissues. Targeting of this metabolic pathway can reduce energy production and cellular proliferation in glioma cells [50]. The endoplasmic reticulum is the predominant site of the elongation of long-chain fatty acids. A study in cultured rat hepatocytes showed that the suppression of N-linked glycosylation enhances the activation of the sterol regulatory element-binding protein-1 following increase in the downstream expression of DNL enzymes, including fatty acid synthase [51].

It should be noticed that the V-type miRNA (Fig. 3b) was expressed regardless of glucose level and may be involved in maintaining constant blood sugar levels. The expression of inverted V-type miRNA was inhibited regardless of glucose level. These results suggest that the target gene is inhibited at the normal glucose level and provide a good basis for future research on miRNAs related to glucose metabolism disorders. By contrast, according to the known T2D-related miRNA target or pathway genes (such as p38, PTEN, PI3K/AKt, Bax, Foxa22, and Glut4) [52, 53], we found that none of these genes were screened probably because the short-term glucose-stimulated culture cannot rapidly induce changes in gene expression.

In this study, we also used the Ago2 IP-seq experiment to observe and reduce the number of possibilities of MTIs of the rat INS-1 pancreatic beta-cells under high glucose concentration. It is noteworthy that in this Ago2 IP-seq experiment, we observed several miRNAs which have been confirmed to be associated with diabetes or metabolic abnormalities, such as miR-30b, miR-143, and miR-27b. miR-30b is one of miRNA which correlates with human obesity and T2D [54]. In addition, miR-143 is correlated with metabolic stress in the liver, resulting in insulin resistance on organismal glucose metabolism [55]. Furthermore, glucocorticoids transcriptionally regulate the miR-27b expression and promote body fat accumulation [56]. In brief, through Ago2 IP-seq, we can observe the changes of MTIs under different experimental conditions accurately and extensively. The identified novel MTIs in this study may provide valuable insights into blood glucose homeostasis.

The two candidate direct target genes (Crem and Fn1) of miR-146b under glucose stimulation are presented in Fig. 7. Notably, the two genes are related to the insulin release mechanism [57], Insulin receptor signaling cascade [58], and the correlation of glycemia and glycosylated hemoglobin among patients with T2D [59], respectively, but no evidence supports that miR-146b can participate in the simultaneous regulation of these genes. Two target genes of miR-146b were still not annotated in miRTarBase [60]; thus, these target genes were potential candidates for experimental validation. According to computational prediction, overexpressed miR-146b mimic, and Ago2IP with qRT-PCR results (Figs. 7 and 8a-c), Crem and Fn1 were highly associated with miR-146b. Further validation by luciferase reporter assay proved that the direct integration between miR-146b and Crem or Fn1 genes (Fig. 8D).

In addition, Yipf3 is a predicted target gene of miR-501-3p and miR-185-5p (Fig. 7). Yipf3 is involved in the maintenance of the Golgi structure and plays a role in hematopoiesis. Due to hyperglycemia, dysregulated hematopoietic cells move to those organs which are affected by diabetes and cause different effects like inflammation, cellular dysfunction, and accelerated apoptosis [61].

## Conclusions

In summary, we combined our computational framework with wet-laboratory experiments to uncover the effect of environmental glucose level on the dynamic of miRNA–mRNA regulatory network in the INS-1 cells. The expression profiles of miRNA and mRNA under various experimental conditions were measured by high-throughput sequencing and the differential expression of the selected genes were validated using qRT-PCR. Our data enabled us to reconstruct the miRNA-mediated regulatory network and disclosed previously unidentified miRNA-mediated regulation in adapting to different glucose levels. Notably, GO analysis demonstrated that genes with similar biological functions were regulated by the same miRNAs and shared similar expression profiles; these findings suggest that miRNA-mediated gene regulation can be used as a biomarker for examination or treatment. Moreover, we found that more than 30% of reported miRNAs associated with diabetes show differential expression with the change in glucose level. In addition, using Ago2 IP experiment and luciferase reporter assay, we showed that miR-146b can inhibit the mRNA levels of Crem and Fn1 genes under high glucose condition and these results provide the first evidence of the involvement of miR-146b in the glucose-stimulated insulin secretion mechanism. These findings offer a potentially significant effect on the understanding of miRNA-mediated regulatory networks in the development and progression of pancreatic diabetes. The integration of experimentally verified data with this computational framework can effectively and efficiently extract the miRNA network and is thus suitable for exploring potential pancreatic disease-associated miRNA and their target genes.

## Methods

### Cell culture, RNA isolation, and library preparation for RNA and small RNA sequencing

INS-1 cells (AddexBio, Taoyuan, Taiwan) were routinely cultured in RPMI-1640 (Thermo Fisher, Waltham, MA, USA) supplemented with 10% FBS, 1 mM pyruvate, 10 mM HEPES, and 50 mM 2-mercaptoethanol at 37 °C in a humidified atmosphere at 5% CO_2_. The cells were detached using Trypsin–EDTA and were passaged once per week. For RNA isolation, INS-1 cells were plated at a density of 10^5^ cells per T-75 flask and incubated in RPMI-1640 containing 2 mM (low glucose), 11.1 mM (moderate glucose), and 30 mM glucose (high glucose). As osmolality control, mannose was added to the culture medium to avoid osmotic effects resulting from low glucose levels [62]. On the following day, the cells were collected, and RNA extraction was carried out. mRNA from the INS-1 cells was extracted using RNAZol kit (SigmaAldrich, Merck KGaA), and miRNA was extracted using miRNeasy Mini Kit (Qiagen, Hilden, Germany). The extracted RNA was dissolved in RNase-free water and stored at − 80 °C. The concentration of RNA was measured using a Nanodrop spectrophotometer (Thermo Fisher, Waltham, MA, USA). The integrity of total RNA was assessed using Agilent 2200 TapeStation-RNA R6K assay (Agilent, Santa Clara, CA). RNA-seq and small RNA-seq cDNA libraries were constructed following the standard Illumina (Illumina, San Diego, CA) protocol. All resulting cDNA library fragments were validated using Agilent 2200 TapeStation-D1000 assay (Agilent). The cDNA libraries were measured using quantitative reverse-transcription PCR (qRT-PCR) (Roche, LightCycler® 480 system, Basel, Switzerland) and a Qubit fluorometer (Invitrogen, Carlsbad, California, USA), pooled, and sequenced on an Illumina NextSeq 500 platform in single-ended mode with 75 bps. Depth of coverage of approximately 20 million or 5 million reads was obtained in RNA-seq or small RNA-seq, respectively. The expression levels of selected target genes in RNA sequencing samples and other independent samples were validated via qRT-PCR method using a general SYBR Green PCR Kit (Roche).

### RNA sequencing data analysis

Quality control of RNA-seq reads was conducted by using the FASTX-Toolkit version 0.0.13.2. Nucleotides with high-quality base calling (Phred quality score ≥ 20, which represents 99% accuracy of a base call) were included, and RNA reads exceeding 35 nucleotides were retained. Then, the reads were mapped to the UCSC *Rattus norvegicus* genome rn6 by using TopHat2 [63] version 2.1.0 with the option --b2-very-sensitive and –G for transcriptome annotations from the UCSC Genome Browser [64]. The transcripts were assembled, and the estimated abundances were normalized by Cufflinks version 2.2.1 [65]. Gene expression profiles were estimated using Cuffdiff version 2.2.1. Genes with FPKM values of less than 10 in both compared samples were filtered. Genes were identified as upregulated or downregulated if the fold change exceeded 1.2 or was less than 0.83, respectively.

### Small RNA sequencing data analysis

The 3′-adapters of the small RNA-seq reads were trimmed using Cutadapt v1.5 [66]. Quality control and data preprocessing were done using the FASTX-Toolkit. Only nucleotides with Phred quality score ≥ 20 and reads longer than 18 were included. To assess the quality of the small RNA distribution and miRNA in the sequencing data, ncPRO-seq [67] package (version 1.6.1) was used to confirm the read distribution in the reference genome. Only reads that were mapped with a maximum of one mismatch and 20 locations in the genome were used (Bowtie [68] v1.1.2 parameter: -v1 -a -m20 --best --strata --nomaqround -f -y). Five databases, namely, the UCSC reference genome (rn5), miRBase v21, UCSC refGene, RFam v11.0, and UCSC RepeatMasker (rn5) were employed for annotation. To quantify the miRNA profiles, miRDeep2 [69] package (version 2.0.0.5) and same bowtie parameter as ncPRO-seq were used. To standardize miRNA expression across different samples, we normalized the read count in each sample into reads per million (RPM). To categorize extremely low-expressed miRNA, we filtered miRNAs with RPM lower than 10 in both compared samples. miRNAs were identified as upregulated or downregulated if the fold change exceeded 1.4 or was less than 0.71, respectively.

### A computational approach for reconstruction of miRNA-mediated gene regulatory network

An overview of the proposed computational approach to reconstruct the miRNA-gene regulatory network is shown in Fig. 1. Information on TF-miRNA regulation was derived from the TransmiR [70] database and review of the literature. DEGs and miRNAs were obtained from expression profiles. Experimental MTIs were obtained from miRTarBase [71]. The predicted MTIs were obtained using four prediction tools, namely, miRanda [72], PITA [73], TargetScan [74], and RNAhybrid [75]. Only MTIs with minimum free energy ≤10 and miRanda score ≥ 140 and predicted by at least three tools were selected. For each MTI, the miRNA and its target gene with inverse expression level (upregulated genes with downregulated miRNAs or downregulated genes with upregulated miRNAs) were selected to further reconstruct the miRNA-mediated regulatory network. Each type of miRNA and its target interaction was based on inverse expression level, whereas each type of TF and its potential association with miRNA were defined on the basis of regulatory type by TransmiR or by the literature. Cytoscape (version 3.4.0) software [76] was used to visualize the association of TF and miRNA or MTI. To discuss the function of the miRNA regulatory network, we further analyzed the miRNA target gene functional annotation by using DAVID [77].

### Argonaute 2 immunoprecipitation sequencing (Ago2 IP-seq)

The detailed information of cell crosslinking, cell lysis and Ago2 IP are described in the Supplementary Information. Purified total RNA from INS-1 cell lysate was used as a template for subsequent RNA-seq and small RNA-seq experiments. The mRNAs with FPKM values of less than 8 and the miRNAs with RPM lower than 100 were filtered. The expression of target genes of miRNA was analyzed using qRT-PCR.

### Experimental validation of miR-146b targets

To investigate the regulation of target genes by miR-146b, we overexpressed the miR-146b, and the levels of expression of two target genes Crem and Fn1 were measured using qRT-PCR. Prior to qRT-PCR, INS-1 cells were transfected with the miR-146b mimics and scrambled control. After 48 h of transfection, total RNA was isolated from the INS-1 cell line, and the mRNA expression levels of four target genes were measured. In addition, the RNA fractions were concentrated using the Ago2 IP method (Supplementary Information) and used to validate the target mRNA of miR-146b by using qRT-PCR.

### Plasmid construction and dual luciferase reporter assay

The 3’UTR of Crem and Fn1 were amplified using genomic DNA and cloned downstream of the Renilla luciferase open reading frame in the pmiRGLO vector (Promega) using PmeI and XbaI restriction sites. The primers for 3’UTR amplification are Crem-F: 5’CTAGCTAGCTAGAGAATAGCCTGACACGGCTA3’; Crem-R: 5’TGCTCTAGAGCAAACTCATTCCAGATAATAA3’;Fn1-F:5’CTAGCTAGCTAGCAGCCCAAGCCAACAAGTGT3’; Fn1-R: TGCTCTAGAGCAAGACAATAATACTGAGCAGT3’. The day before performing the luciferase reporter assay, 12 well plate with 1 × 10^4^ INS1 cells were co-transfected with either empty luciferase vector or the construct containing 3’UTR of two target genes Crem and Fn1 and miR-146b mimics or scramble control. pmiRGLO vector with no insert was used as control. The plates were removed from the incubator next day and 200 μl of Dual-Glo reagent was added to each well and mixed and allowed to wait for 10 min for cell lysis to occur, then firefly luminescence was measured using Lumat 9507 LB (Berthold Technologies). One hundred microliter of Dual-Glo Stop & Glo was added to the cell lysate and mixed well and then renilla luminescence was measured. Normalized luciferase activity (firefly luciferase activity/renilla luciferase activity) for each construct was calculated.

## Supplementary information

**Additional file 1.** RNA-seq data.

**Additional file 2.** sRNA-seq data.

**Additional file 3.** Ago-IP seq data.

**Additional file 4.** Supplementary Information.

## Data Availability

The additional files and supplementary information mentioned in the article are all availability of data and materials within this study.

## Abbreviations

miRNAs: MicroRNAs
RNA-seq: RNA sequencing
sRNA-seq: Small RNA sequencing
DEmiRs: Differentially expressed miRNAs
Ago IP-seq: Argonaute immunoprecipitation sequencing
CLIP-seq: Cross-link immunoprecipitation sequencing
ChIP-seq: Chromatin immunoprecipitation sequencing
MTIs: MiRNA–target interactions
PI3K: Phosphatidylinositol 3-kinase
DEGs: Differentially expressed genes
src: Scrambled control

## References

[1] Bartel DP (2004). MicroRNAs: genomics, biogenesis, mechanism, and function. Cell.

[2] Carleton M, Cleary MA, Linsley PS (2007). MicroRNAs and cell cycle regulation. Cell Cycle.

[3] Harfe BD (2005). MicroRNAs in vertebrate development. Curr Opin Genet Dev.

[4] Lynam-Lennon N, Maher SG, Reynolds JV (2009). The roles of microRNA in cancer and apoptosis. Biol Rev Camb Philos Soc.

[5] Soifer HS, Rossi JJ, Saetrom P (2007). MicroRNAs in disease and potential therapeutic applications. Mol Ther.

[6] Chou CH, Shrestha S, Yang CD, Chang NW, Lin YL, Liao KW, Huang WC, Sun TH, Tu SJ, Lee WH, et al. miRTarBase update 2018: a resource for experimentally validated microRNA-target interactions. Nucleic Acids Res. 2017;4;46(D1):D296-302. 10.1093/nar/gkx1067 PMC5753222 29126174

[7] Schuit FC, Huypens P, Heimberg H, Pipeleers DG (2001). Glucose sensing in pancreatic beta-cells: a model for the study of other glucose-regulated cells in gut, pancreas, and hypothalamus. Diabetes.

[8] Paris M, Bernard-Kargar C, Berthault MF, Bouwens L, Ktorza A (2003). Specific and combined effects of insulin and glucose on functional pancreatic beta-cell mass in vivo in adult rats. Endocrinology.

[9] Heit JJ, Karnik SK, Kim SK (2006). Intrinsic regulators of pancreatic beta-cell proliferation. Annu Rev Cell Dev Biol.

[10] Wu L, Dai X, Zhan J, Zhang Y, Zhang H, Zhang H, Zeng S, Xi W (2015). Profiling peripheral microRNAs in obesity and type 2 diabetes mellitus. APMIS.

[11] Bai C, Li X, Gao Y, Wang K, Fan Y, Zhang S, Ma Y, Guan W (2016). Role of microRNA-21 in the formation of insulin-producing cells from pancreatic progenitor cells. Biochim Biophys Acta.

[12] Floris I, Descamps B, Vardeu A, Mitic T, Posadino AM, Shantikumar S, Sala-Newby G, Capobianco G, Mangialardi G, Howard L (2015). Gestational diabetes mellitus impairs fetal endothelial cell functions through a mechanism involving microRNA-101 and histone methyltransferase enhancer of zester homolog-2. Arterioscler Thromb Vasc Biol.

[13] Poy MN, Eliasson L, Krutzfeldt J, Kuwajima S, Ma X, Macdonald PE, Pfeffer S, Tuschl T, Rajewsky N, Rorsman P (2004). A pancreatic islet-specific microRNA regulates insulin secretion. Nature.

[14] Avnit-Sagi T, Kantorovich L, Kredo-Russo S, Hornstein E, Walker MD (2009). The promoter of the pri-miR-375 gene directs expression selectively to the endocrine pancreas. PLoS One.

[15] El Ouaamari A, Baroukh N, Martens GA, Lebrun P, Pipeleers D, van Obberghen E (2008). miR-375 targets 3′-phosphoinositide-dependent protein kinase-1 and regulates glucose-induced biological responses in pancreatic beta-cells. Diabetes.

[16] Hashimoto N, Kido Y, Uchida T, Asahara S, Shigeyama Y, Matsuda T, Takeda A, Tsuchihashi D, Nishizawa A, Ogawa W (2006). Ablation of PDK1 in pancreatic beta cells induces diabetes as a result of loss of beta cell mass. Nat Genet.

[17] Walker MD (2008). Role of MicroRNA in pancreatic beta-cells: where more is less. Diabetes.

[18] LaPierre MP, Stoffel M (2017). MicroRNAs as stress regulators in pancreatic beta cells and diabetes. Mol Metab.

[19] Keller DM, Clark EA, Goodman RH (2012). Regulation of microRNA-375 by cAMP in pancreatic beta-cells. Mol Endocrinol.

[20] Wang Y, Liu J, Liu C, Naji A, Stoffers DA (2013). MicroRNA-7 regulates the mTOR pathway and proliferation in adult pancreatic beta-cells. Diabetes.

[21] Nieto M, Hevia P, Garcia E, Klein D, Alvarez-Cubela S, Bravo-Egana V, Rosero S, Damaris Molano R, Vargas N, Ricordi C (2012). Antisense miR-7 impairs insulin expression in developing pancreas and in cultured pancreatic buds. Cell Transplant.

[22] Baroukh N, Ravier MA, Loder MK, Hill EV, Bounacer A, Scharfmann R, Rutter GA, Van Obberghen E (2007). MicroRNA-124a regulates Foxa2 expression and intracellular signaling in pancreatic beta-cell lines. J Biol Chem.

[23] Wang P, Chen L, Zhang J, Chen H, Fan J, Wang K, Luo J, Chen Z, Meng Z, Liu L (2014). Methylation-mediated silencing of the miR-124 genes facilitates pancreatic cancer progression and metastasis by targeting Rac1. Oncogene.

[24] Yan Z, Shah PK, Amin SB, Samur MK, Huang N, Wang X, Misra V, Ji H, Gabuzda D, Li C (2012). Integrative analysis of gene and miRNA expression profiles with transcription factor-miRNA feed-forward loops identifies regulators in human cancers. Nucleic Acids Res.

[25] Huang GT, Athanassiou C, Benos PV (2011). mirConnX: condition-specific mRNA-microRNA network integrator. Nucleic Acids Res.

[26] Bisognin A, Sales G, Coppe A, Bortoluzzi S, Romualdi C (2012). MAGIA(2): from miRNA and genes expression data integrative analysis to microRNA-transcription factor mixed regulatory circuits (2012 update). Nucleic Acids Res.

[27] Hsu SD, Huang HY, Chou CH, Sun YM, Hsu MT, Tsou AP (2015). Integrated analyses to reconstruct microRNA-mediated regulatory networks in mouse liver using high-throughput profiling. BMC Genomics.

[28] Gosline SJ, Gurtan AM, JnBaptiste CK, Bosson A, Milani P, Dalin S, Matthews BJ, Yap YS, Sharp PA, Fraenkel E (2016). Elucidating MicroRNA regulatory networks using transcriptional, post-transcriptional, and histone modification measurements. Cell Rep.

[29] Skelin M, Rupnik M, Cencic A (2010). Pancreatic beta cell lines and their applications in diabetes mellitus research. ALTEX.

[30] Kanematsu S, Tanimoto K, Suzuki Y, Sugano S (2014). Screening for possible miRNA-mRNA associations in a colon cancer cell line. Gene.

[31] Asfari M, Janjic D, Meda P, Li G, Halban PA, Wollheim CB (1992). Establishment of 2-mercaptoethanol-dependent differentiated insulin-secreting cell lines. Endocrinology.

[32] Andrali SS, Sampley ML, Vanderford NL, Ozcan S (2008). Glucose regulation of insulin gene expression in pancreatic beta-cells. Biochem J.

[33] Esguerra JL, Mollet IG, Salunkhe VA, Wendt A, Eliasson L (2014). Regulation of pancreatic Beta cell stimulus-secretion coupling by microRNAs. Genes (Basel).

[34] Chakraborty C, Doss CG, Bandyopadhyay S, Agoramoorthy G (2014). Influence of miRNA in insulin signaling pathway and insulin resistance: micro-molecules with a major role in type-2 diabetes. Wiley Interdiscip Rev RNA.

[35] Regazzi R, Rodriguez-Trejo A, Jacovetti C (2016). Insulin secretion in health and disease: nutrients dictate the pace. Proc Nutr Soc.

[36] Salunkhe VA, Esguerra JL, Ofori JK, Mollet IG, Braun M, Stoffel M, Wendt A, Eliasson L (2015). Modulation of microRNA-375 expression alters voltage-gated Na(+) channel properties and exocytosis in insulin-secreting cells. Acta Physiol (Oxf).

[37] Sebastiani G, Po A, Miele E, Ventriglia G, Ceccarelli E, Bugliani M, Marselli L, Marchetti P, Gulino A, Ferretti E (2015). MicroRNA-124a is hyperexpressed in type 2 diabetic human pancreatic islets and negatively regulates insulin secretion. Acta Diabetol.

[38] Shaer A, Azarpira N, Karimi MH, Soleimani M, Dehghan S. Differentiation of human-induced pluripotent stem cells into insulin-producing clusters by MicroRNA-7. Exp Clin Transplant. 2016;14(5):555-63. 10.6002/ect.2014.0144 26103160

[39] Xu H, Abuhatzira L, Carmona GN, Vadrevu S, Satin LS, Notkins AL (2015). The Ia-2beta intronic miRNA, miR-153, is a negative regulator of insulin and dopamine secretion through its effect on the Cacna1c gene in mice. Diabetologia.

[40] Taubes G (2008). Diabetes. Paradoxical effects of tightly controlled blood sugar. Science.

[41] Winter T (2002). Increased blood sugar in type 2 diabetes mellitus: what now?. Praxis (Bern 1994).

[42] XG HK, Grayson TB, Shalev A (2016). Cytokines Regulate β-Cell Thioredoxin-interacting Protein (TXNIP) via Distinct Mechanisms and Pathways. J Biol Chem.

[43] Zhu H, Leung SW (2015). Identification of microRNA biomarkers in type 2 diabetes: a meta-analysis of controlled profiling studies. Diabetologia.

[44] Simpson K, Wonnacott A, Fraser DJ, Bowen T (2016). MicroRNAs in diabetic nephropathy: from biomarkers to therapy. Curr Diab Rep.

[45] Belgardt BF, Ahmed K, Spranger M, Latreille M, Denzler R, Kondratiuk N, von Meyenn F, Villena FN, Herrmanns K, Bosco D (2015). The microRNA-200 family regulates pancreatic beta cell survival in type 2 diabetes. Nat Med.

[46] Delic D, Eisele C, Schmid R, Luippold G, Mayoux E, Grempler R. Characterization of Micro-RNA changes during the 652 progression of type 2 diabetes in Zucker Diabetic fatty rats. Int J Mol Sci. 2016;17(5):665. 10.3390/ijms17050665 PMC4881491 27153060

[47] Bakan E, Yildirim A, Kurtul N, Polat MF, Dursun H, Cayir K (2006). Effects of type 2 diabetes mellitus on plasma fatty acid composition and cholesterol content of erythrocyte and leukocyte membranes. Acta Diabetol.

[48] Russell DW, Halford RW, Ramirez DM, Shah R, Kotti T (2009). Cholesterol 24-hydroxylase: an enzyme of cholesterol turnover in the brain. Annu Rev Biochem.

[49] Ahn BY, Saldanha-Gama RF, Rahn JJ, Hao X, Zhang J, Dang NH, Alshehri M, Robbins SM, Senger DL (2016). Glioma invasion mediated by the p75 neurotrophin receptor (p75(NTR)/CD271) requires regulated interaction with PDLIM1. Oncogene.

[50] Lin H, Patel S, Affleck VS, Wilson I, Turnbull DM, Joshi AR, Maxwell R, Stoll EA. Fatty acid oxidation is required for the respiration and proliferation of malignant glioma cells. Neuro Oncol. 2017;19(1):43-54. 10.1093/neuonc/now128 PMC5193020 27365097

[51] Sanders FW, Griffin JL (2016). De novo lipogenesis in the liver in health and disease: more than just a shunting yard for glucose. Biol Rev Camb Philos Soc.

[52] Gong W, Xiao D, Ming G, Yin J, Zhou H, Liu Z (2014). Type 2 diabetes mellitus-related genetic polymorphisms in microRNAs and microRNA target sites. J Diabetes.

[53] Guay C, Roggli E, Nesca V, Jacovetti C, Regazzi R (2011). Diabetes mellitus, a microRNA-related disease?. Transl Res.

[54] Hu F, Wang M, Xiao T, Yin B, He L, Meng W, Dong M (2015). Liu F: miR-30 promotes thermogenesis and the development of beige fat by targeting RIP140. Diabetes.

[55] Jordan SD, Kruger M, Willmes DM, Redemann N, Wunderlich FT, Bronneke HS, Merkwirth C, Kashkar H, Olkkonen VM, Bottger T (2011). Obesity-induced overexpression of miRNA-143 inhibits insulin-stimulated AKT activation and impairs glucose metabolism. Nat Cell Biol.

[56] Kong X, Yu J, Bi J, Qi H, Di W, Wu L, Wang L, Zha J, Lv S, Zhang F (2015). Glucocorticoids transcriptionally regulate miR-27b expression promoting body fat accumulation via suppressing the browning of white adipose tissue. Diabetes.

[57] Favre D, Niederhauser G, Fahmi D, Plaisance V, Brajkovic S, Beeler N, Allagnat F, Haefliger JA, Regazzi R, Waeber G (2011). Role for inducible cAMP early repressor in promoting pancreatic beta cell dysfunction evoked by oxidative stress in human and rat islets. Diabetologia.

[58] Benyoucef S, Surinya KH, Hadaschik D, Siddle K (2007). Characterization of insulin/IGF hybrid receptors: contributions of the insulin receptor L2 and Fn1 domains and the alternatively spliced exon 11 sequence to ligand binding and receptor activation. Biochem J.

[59] Lopez JJ, Jardin I, Chamorro CC, Duran ML, Tarancon Rubio MJ, Reyes Panadero M, Jimenez F, Montero R, Gonzalez MJ, Martinez M, et al. Involvement of stanniocalcins in the deregulation of glycaemia in obese mice and type 2 diabetic patients. J Cell Mol Med. 2018;22(1):684–94. 10.1111/jcmm.13355 PMC5742690 28990324

[60] Chou CH, Chang NW, Shrestha S, Hsu SD, Lin YL, Lee WH, Yang CD, Hong HC, Wei TY, Tu SJ, et al. miRTarBase 2016:updates to the experimentally validated miRNA-target interactions database. Nucleic Acids Res. 2016;4;44(D1):D239-47. 10.1093/nar/gkv1258 PMC4702890 26590260

[61] Kojima H, Kim J, Chan L (2014). Emerging roles of hematopoietic cells in the pathobiology of diabetic complications. Trends Endocrinol Metab.

[62] Wu L, Derynck R (2009). Essential role of TGF-beta signaling in glucose-induced cell hypertrophy. Dev Cell.

[63] Kim D, Pertea G, Trapnell C, Pimentel H, Kelley R, Salzberg SL (2013). TopHat2: accurate alignment of transcriptomes in the presence of insertions, deletions and gene fusions. Genome Biol.

[64] Karolchik D, Baertsch R, Diekhans M, Furey TS, Hinrichs A, Lu Y, Roskin KM, Schwartz M, Sugnet CW, Thomas DJ (2003). The UCSC genome browser database. Nucleic Acids Res.

[65] Trapnell C, Hendrickson DG, Sauvageau M, Goff L, Rinn JL, Pachter L (2013). Differential analysis of gene regulation at transcript resolution with RNA-seq. Nat Biotechnol.

[66] Martin M (2011). Cutadapt removes adapter sequences from high-throughput sequencing reads. EMBnet J.

[67] Chen CJ, Servant N, Toedling J, Sarazin A, Marchais A, Duvernois-Berthet E, Cognat V, Colot V, Voinnet O (2012). Heard E *et al*: ncPRO-seq: a tool for annotation and profiling of ncRNAs in sRNA-seq data. Bioinformatics.

[68] Langmead B, Trapnell C, Pop M, Salzberg SL (2009). Ultrafast and memory-efficient alignment of short DNA sequences to the human genome. Genome Biol.

[69] Friedlander MR, Mackowiak SD, Li N, Chen W (2012). Rajewsky N: miRDeep2 accurately identifies known and hundreds of novel microRNA genes in seven animal clades. Nucleic Acids Res.

[70] Wang J, Lu M, Qiu C, Cui Q (2010). TransmiR: a transcription factor-microRNA regulation database. Nucleic Acids Res.

[71] Chou CH, Chang NW, Shrestha S, Hsu SD, Lin YL, Lee WH, Yang CD, Hong HC, Wei TY (2016). Tu SJ *et al*: miRTarBase 2016: updates to the experimentally validated miRNA-target interactions database. Nucleic Acids Res.

[72] Enright AJ, John B, Gaul U, Tuschl T, Sander C, Marks DS (2003). MicroRNA targets in drosophila. Genome Biol.

[73] Kertesz M, Iovino N, Unnerstall U, Gaul U, Segal E (2007). The role of site accessibility in microRNA target recognition. Nat Genet.

[74] Lewis BP, Burge CB, Bartel DP (2005). Conserved seed pairing, often flanked by adenosines, indicates that thousands of human genes are microRNA targets. Cell.

[75] Kruger J, Rehmsmeier M (2006). RNAhybrid: microRNA target prediction easy, fast and flexible. Nucleic Acids Res.

[76] Shannon P, Markiel A, Ozier O, Baliga NS, Wang JT, Ramage D, Amin N, Schwikowski B, Ideker T (2003). Cytoscape: a software environment for integrated models of biomolecular interaction networks. Genome Res.

[77] Huang da W, Sherman BT, Lempicki RA (2009). Systematic and integrative analysis of large gene lists using DAVID bioinformatics resources. Nat Protoc.

