# Supplementary material for: Multi-omics profiling reveals microRNA-mediated insulin signaling networks
Source: BMC Bioinformatics. 2020 Sep 17;21(Suppl 13):389. doi: 10.1186/s12859-020-03678-0 (PMC7496206; doi:10.1186/s12859-020-03678-0)
Supplement: Supplementary file 4 — Additional file 4. Supplementary Information. [file 12859_2020_3678_MOESM4_ESM.docx]

**Supplementary Information**

**Supplementary Materials and Methods**

**Glucose effects on INS-1 cell morphology**

In order to observe cell morphology, INS-1 cells were seeded at a density of 2 × 10^4^ cells and were treated with 2 mM (low), 11.1 mM (moderate), and 30 mM (high) glucose concentrations. The cells were allowed to grow for four days and monitored carefully and the images were taken at different time periods and glucose concentrations.

**Measurement for growth curve and cell doubling time**

A total of 2 × 10^4^ cells were cultured in 2 mM, 11.1 mM, and 30 mM glucose levels for four days to measure their growth curves and cell doubling time. Mannose was added to the culture solution when the glucose level was lower than normal for osmoregulation and to avoid effects of osmotic pressure resulting from low glucose levels.

**UV-crosslinking, cell lysis and Ago-Immunoprecipitation (IP)**

INS-1 cells were grown to 80-100% confluence for Ago-immunoprecipitation (IP). A 10 cm dish was placed on bed of ice and rinsed once with 3 mL cold PBS. PBS was discarded and another 3 mL cold PBS was added. While keeping the dish on bed of ice, the lid was taken off and irradiated with UV. A 400 mJ/cm^2^ of constant energy was used to crosslink 80-100% confluent dish. The cells were scrapped off and collected by washing with PBS. The cells were spun at 1000 rpm for 10 min at 4^o^C and the pellets were collected. Excessive PBS was discarded and the pellet was re-suspended in 1 mL cold PBS and transferred to RNAase free epi tube. The epi tube was quickly spun at 18000xg for a min to pellet the cells followed by discarding 900 μL of PBS and leaving 100 μL along with the pellet. The pellet was snap frozen in liquid nitrogen or on ethanol-dry ice bath and stored at -80^o^C or proceeded to cell lysis.

Whole cell lysates were collected with 1 mL lysis buffer (50 mM Tris-HCl, pH 7.4; 100 mM NaCl; 1% NP-40; 0.1% SDS; 0.5% sodium deoxycholate) with protease inhibitor (Roche cocktail). Typically, per 2 mg protein, 5 μL antibody (Wako Chemicals: Anti Ago1, Monoclonal Antibody (2A7) | 015-22411; Anti HuAgo2, Monoclonal Antibody | 011-22033, 015-22031), and 30-50 μL Protein G Dynabeads (Invitrogen) were used. Protein G Dynabeads were freshly prepared and washed three times with PBS according to the manufacturer’s instruction. The antibody was then added to Dynabeads in a total volume of 200 μL and incubated on a rotor for 45 min-1 h at 4^o^C. The cell lysates were added to antibody Dynabeads mix and incubated overnight on a rotor at 4^o^C . The magnetic stand (Invitrogen) was used to immobilize the beads and the supernatant was removed. The beads were washed sequentially two times with 900 μL cold High salt wash buffer (50 mM Tris-HCl pH 7.4, 1 M NaCl, 1 mM EDTA, 1% NP-40, 0.1% SDS, 0.5% sodium deoxycholate) and wash buffer (20 mM Tris-HCl pH 7.4, 10 mM MgCl_2_, 0.2% Tween-20). Following the last wash and spin-down, 1 mL of TRIzol reagent (Thermo Fisher) per 2 mg cell lysates or per eppendorf tube was added. A total of 250 μL chloroform was added to each 1 mL of TRIzol and shook vigorously for 15 sec. The mixture was allowed to stand for 3 min and centrifuged at 12,000 rpm for 15 min. The supernatant was removed and 500 μL of isopropanol was added per 1 mL TRIzol. Finally 1 μL glycogen (Thermo Fisher R0561) was added, vortexed and precipitated at -80^o^C overnight.

On next day, the precipitated RNA was centrifuged at 12,000 rpm at 4^o^C for 15-30 min followed by 80 % ethanol wash and centrifugation at 12,000 rpm at 4^o^C for 15 min. The pellet (either visible or invisible) was dissolved with DEPC-treated ddH_2_O. The 260/280 ratio and the RNA concentration were measured prior proceed to subsequent experiments.

**Transfection of miR-146b**

miR-146b-5p mimics (5’UUCCCUUUGUCAUCCUAUGCCU3’) and scrambled control were purchased and prepared according to manufacturer’s instructions (Dharmacon, Thermo Scientific, USA). Day before transfection, 4 x 10^5^ cells per well in a volume of 2 mL were grown in 6 well plates and incubated at 37^o^C and 5% CO_2_. 80 nM of miR-204 mimics and scrambled control were transfected into the INS-1 cells using Turbofect (Dharmacon) Reagent. 24 h post transfection, the cells were harvested for mRNA and miRNA analyses.

**Reverse transcription and quantitative real-time PCR for miRNA**

Total RNA containing miRNA was reverse transcribed using miScript II RT Kit (Qiagen). Briefly, 20 μL reaction mixture was prepared by combining 4 μL 5 x miScript HiSpec Buffer, 2 μL 10 x miScript Nucleics Mix, 2 μL miScript Reverse Transcriptase Mix, 1 μg Template RNA and variable amount of RNase-free water. The mixture was incubated in PCR tube for 60 min at 37^o^C and then inactivated at 95^o^C for 5 min. Real-time PCR for miRNA was performed using miScript SYBR Green PCR Kit (Qiagen) on ABI PRISM® 7000 (Applied Biosystems, Foster City, CA, USA). The miRNA specific primer for miR-146b was 5’UUCAAGUAAUCCAGGAUAGGCU3’. A total reaction volume 20 μL was prepared by mixing 10 μL 2 x QuantiTect SYBR Green PCR Master Mix, 2 μL 10 x miScript Universal Primer, 2 μL 10 x miScript Primer Assay, 200 ng template cDNA and variable amount of RNA free water. The PCR amplification conditions were: initial incubation at 95^o^C for 15 sec, followed by 45 cycles of 94^o^C for 15 sec, 55^o^C for 30 sec and 70^o^C for 30 sec. The expression level of miR-146b was determined relative to U6 miRNA and calculated from Ct values using the ΔΔCT method. All quantitative PCR reactions were performed with six replicates.

**Reverse transcription and quantitative real-time PCR for mRNA**

Total RNA containing mRNA was reverse transcribed using GScript First-Strand Synthesis (GeneDirex) protocol. A reaction mixture of 13 μL was prepared by mixing 1 μL of oligo, 2 μg of template RNA, 1 μL of 10 mM dNTP mix and variable amount of RNase free water. The mixture was incubated at 65^o^C for 5 min and then placed on ice for 1 min. To this mixture, 4 μL of 5 x First Strand Buffer, 1 μL of 0.1M DTT, 1 μL of DEPC water, 1 μL of GScript RTase were added and mixed thoroughly and incubated for 5 min at room temperature. The mixture was then incubated at 50^o^C for 60 min and inactivated at 70^o^C for 15 min.

Real-time PCR was conducted by ABI PRISM® 7000 (Applied Biosystems, Foster City, CA, USA). qRT-PCR of mRNA was performed using SYBR Green PCR Kit (Roche). A total reaction volume 10 μL was prepared by mixing 5 μL SYBR Master mix, 1 μL Forward primer, 1 μL Reverse primer, 50 ng cDNA and variable amount of RNA free water. The PCR amplification steps were: initial incubation at 95^o^C, followed by denaturation at 95^o^C for 15 sec, annealing at 60^o^C for 30 sec and extension at 72^o^C for 30 sec. The relative expression level of mRNA was normalized to GAPDH and was calculated using ΔΔCT method. The primers used in qRT-PCR analysis of mRNA levels for Glp1r forward 5’-ACCACTGCACAGACCCAAGTC-3' and reverse 5'-TCTCCAGCTCTTGGAGTCTTTCTT-3'; Trpc4 forward 5'-CATAGATTATGATTTAAGCCCCACG-3' and reverse 5'-ACACTCCTTCTCCAAGTTGTCACA-3'; Pde4b forward 5'-GGCAATGGACTGATCGCAT-3’ and reverse 5'-CTCCATTCCCCTCTCCCG-3'; Rasgrp2 forward 5'- CAGGTACTGGGTCTCAGCCTTC -3' and reverse 5'- GCTCCTTGATCTGTTCAGCCA -3'; Camk4 forward 5'- AACCCGGCTTCCTTTAGGG -3' and reverse 5'- GCTTCCCCACCTTGCTGAA -3'; Crem forward 5'- TGCCACAAGGTGTGGTGATG -3' and reverse 5'- TGCTTCTTCTGCTAGTTGCTGG -3'; Cited2 5'- ACTTTCTCCCGTGCTCAACTG -3' and reverse 5'- TGAAACCATCTACAAAATCCACAAG -3'; Ins1 5'- CACTTCCTACCCCTGCTGG -3' and reverse 5'- ACCACAAAGATGCTGTTTGACA -3'; Calr 5'- CATGAGCAGAATATCGACTGTGGGGGCGG -3' and reverse 5'- TTTATCCTCGGCCTCCTCTTCCTCT -3'.

**Supplementary Data**

**Supplementary Figures**

**
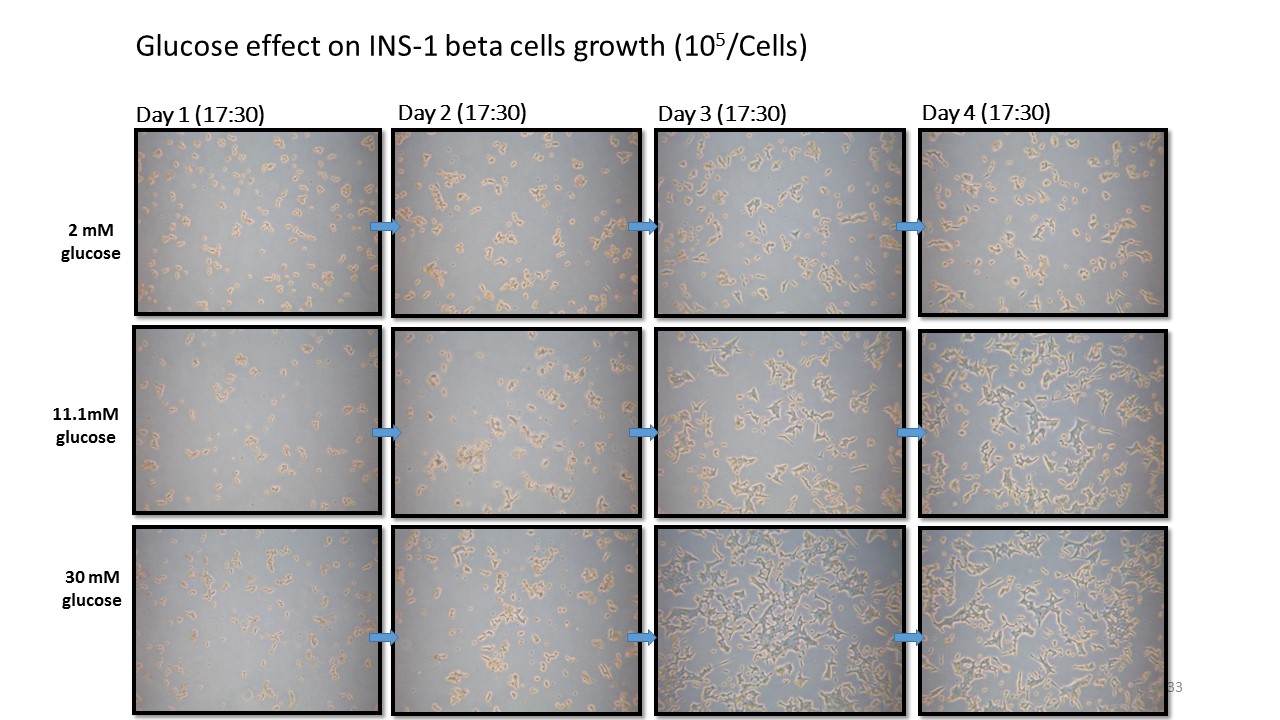
**

**Figure S1**. INS-1 cell morphology in different time periods and different glucose concentrations (cells collected from day 2 experiments were taken for sequencing).

**
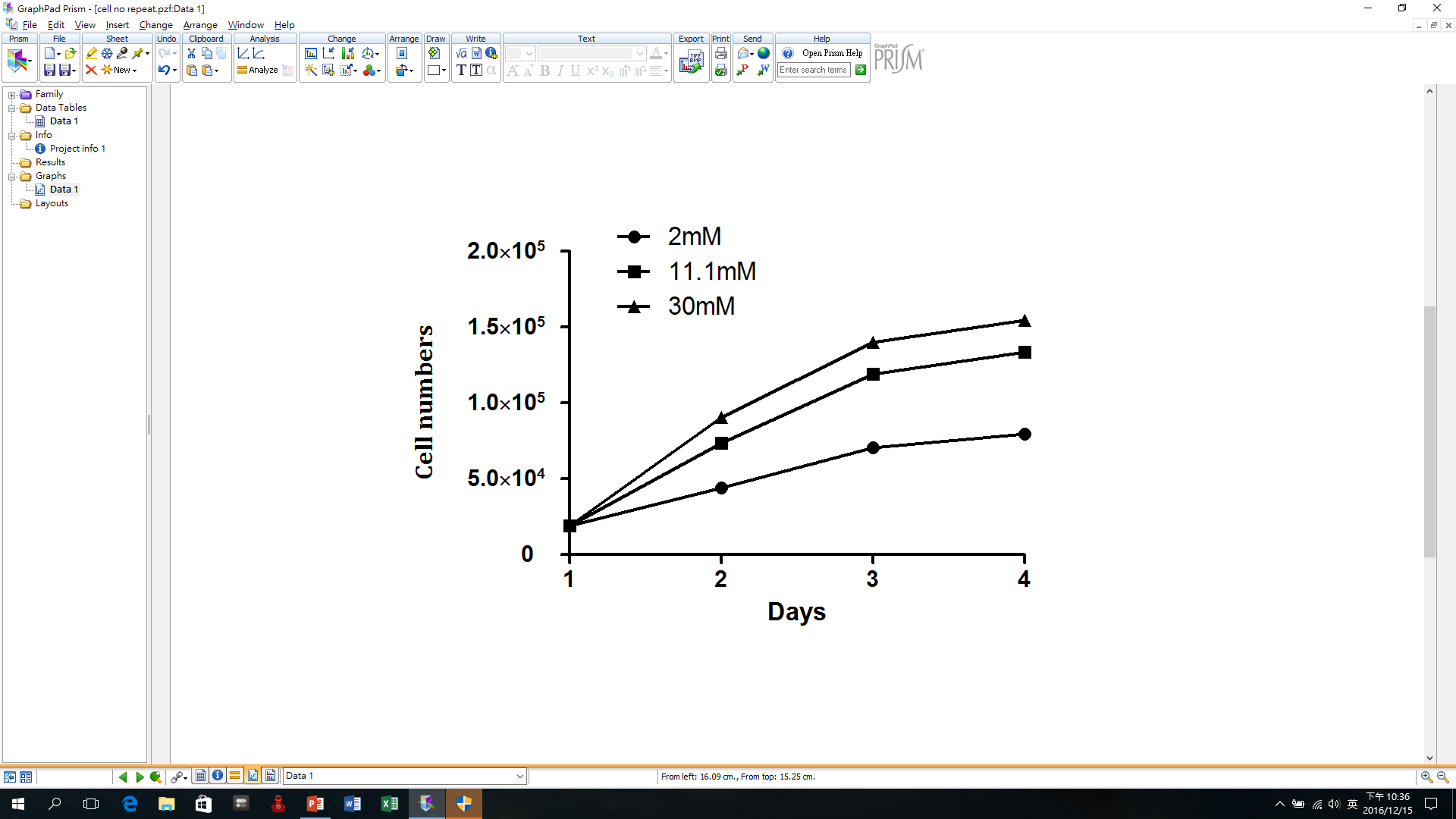
**

**Figure S2.** INS-1 cell growth curve assay. Each point shows number of cells against time.


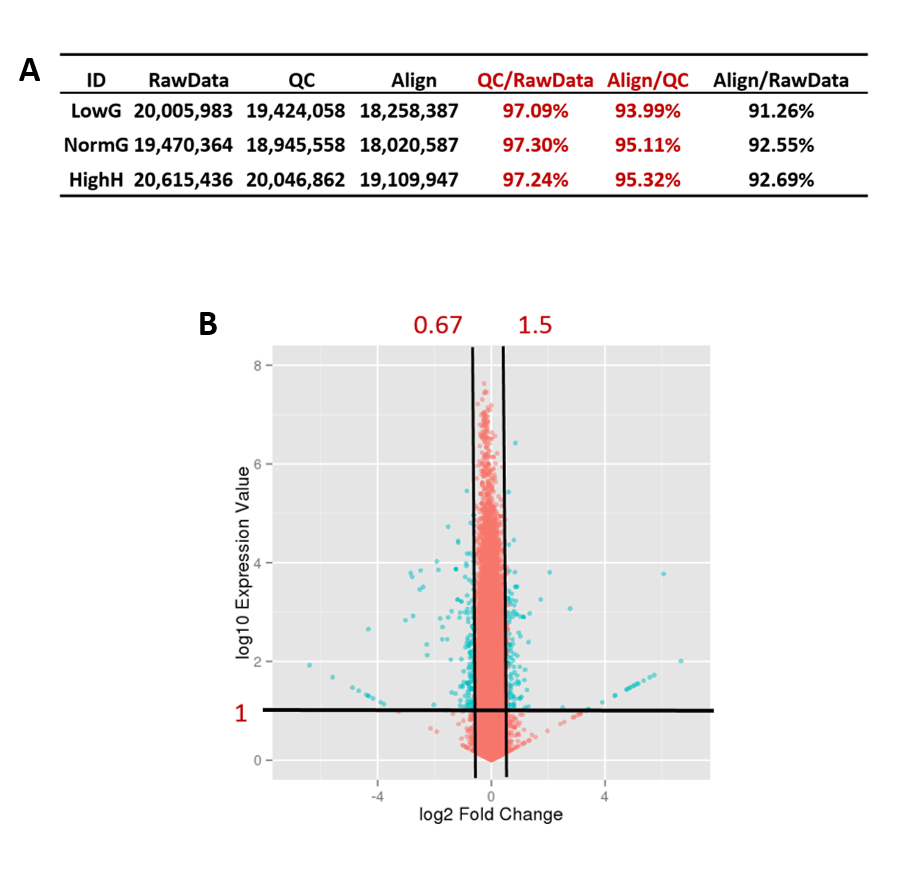


**Figure S3. Read quality and distribution of RNA-seq data.** (A) Summary of the reads mapped to reference genome (B) Volcano plot showing the differentially expressed genes identified by RNA-seq analysis.

**A**

**
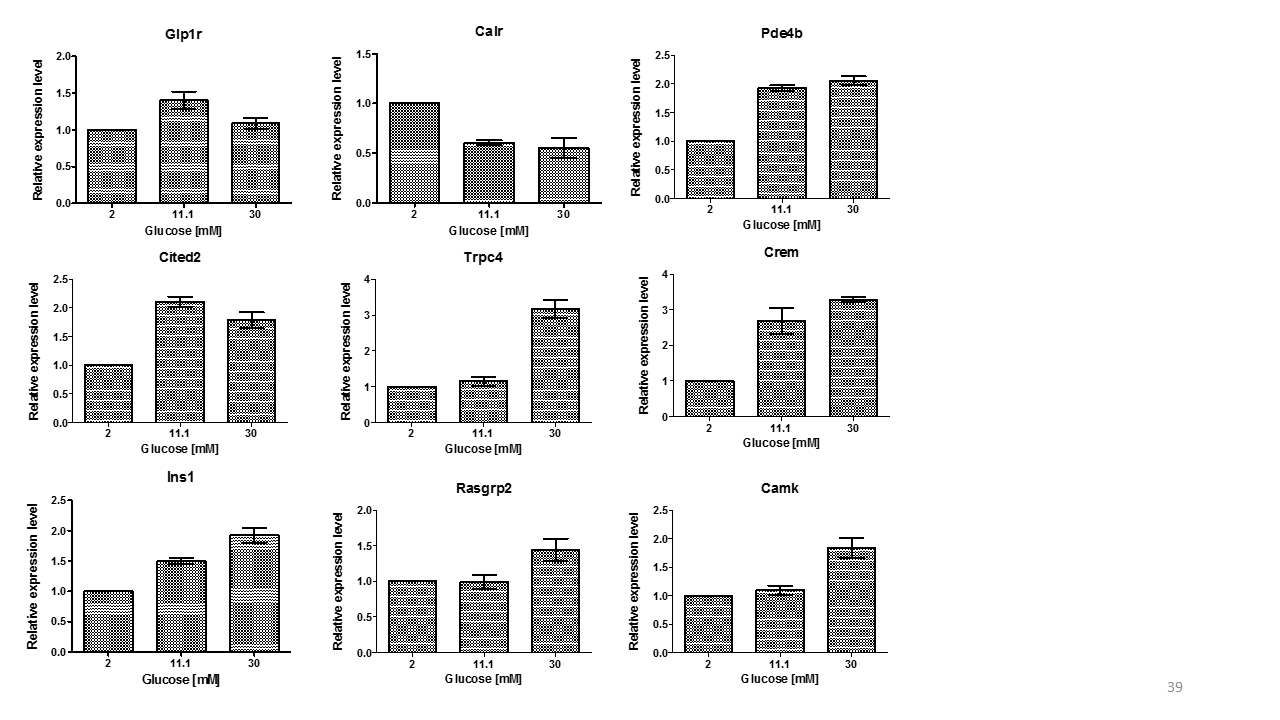
**

**B**

**
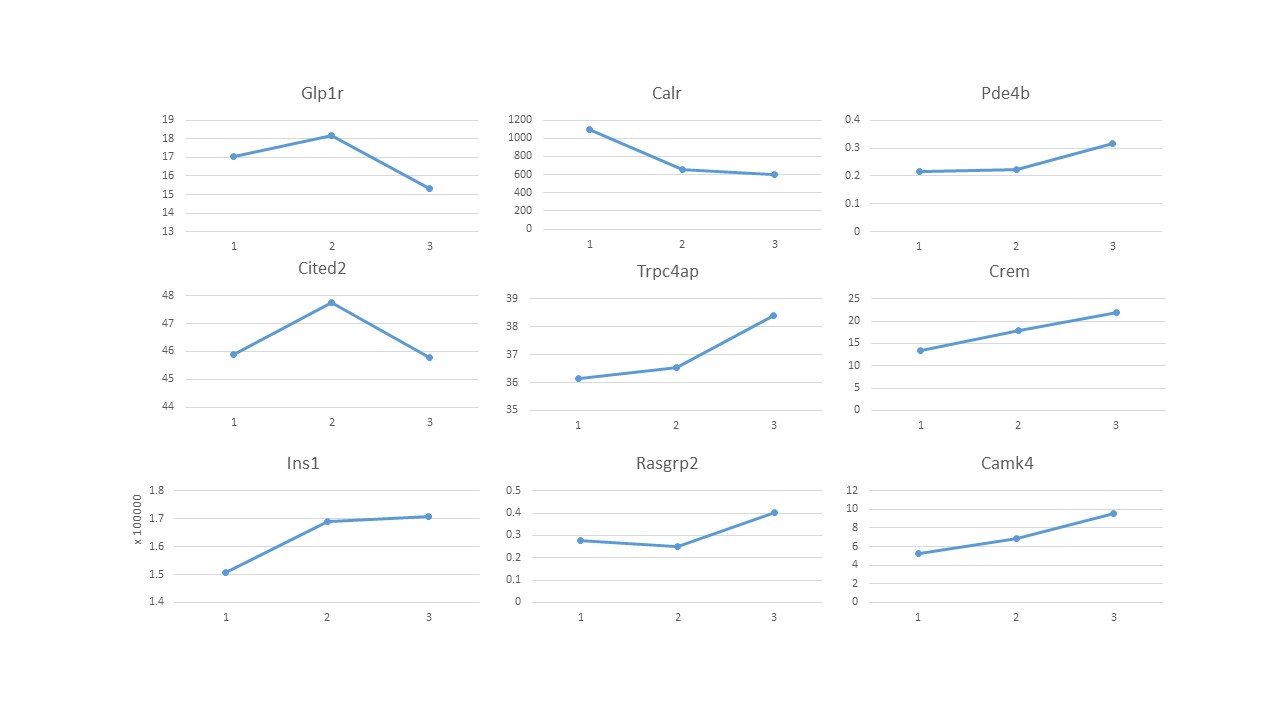
**

**Figure S4. Validation of differential gene expression results obtained from RNA-seq.** (A) Using RNA sequencing samples and other independent samples the expression of selected target genes are validated by quantitative PCR method (B) RNA-seq profiles of the selected target genes showing FPKM value.

**
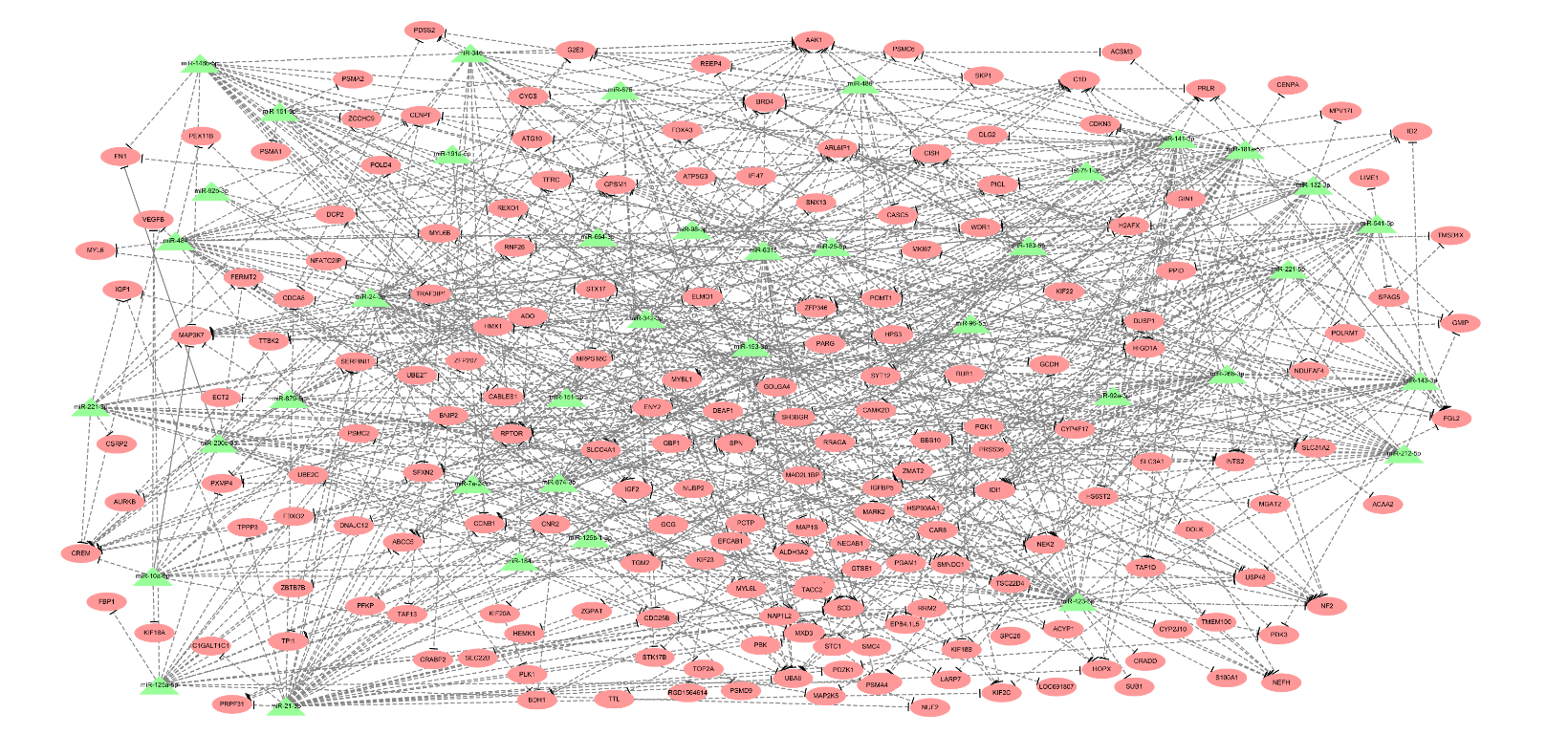
**

**Figure S5.** The DN-miRNA-mediated gene regulatory network in low-glucose concentration.

**
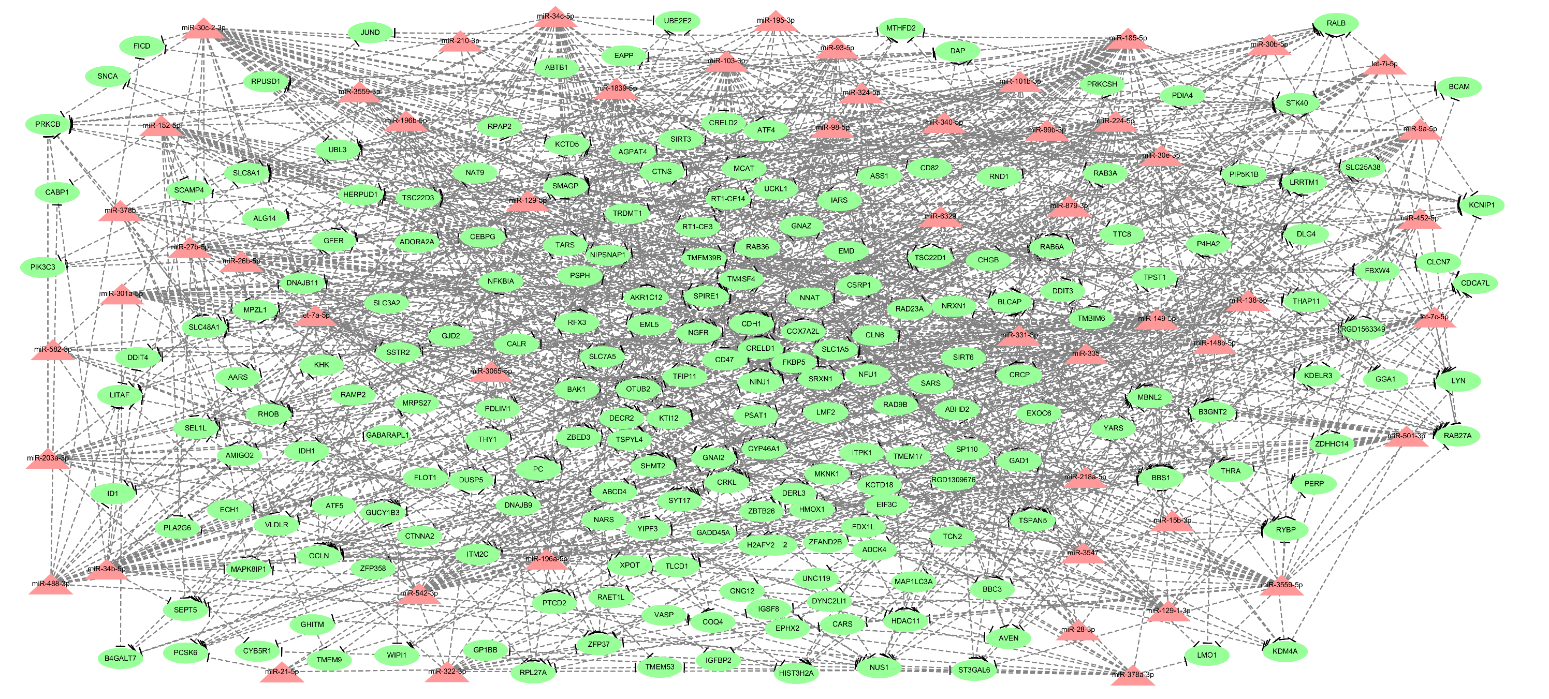
**

**Figure S6.** The UP-miRNA-mediated gene regulatory network in low-glucose concentration.

**
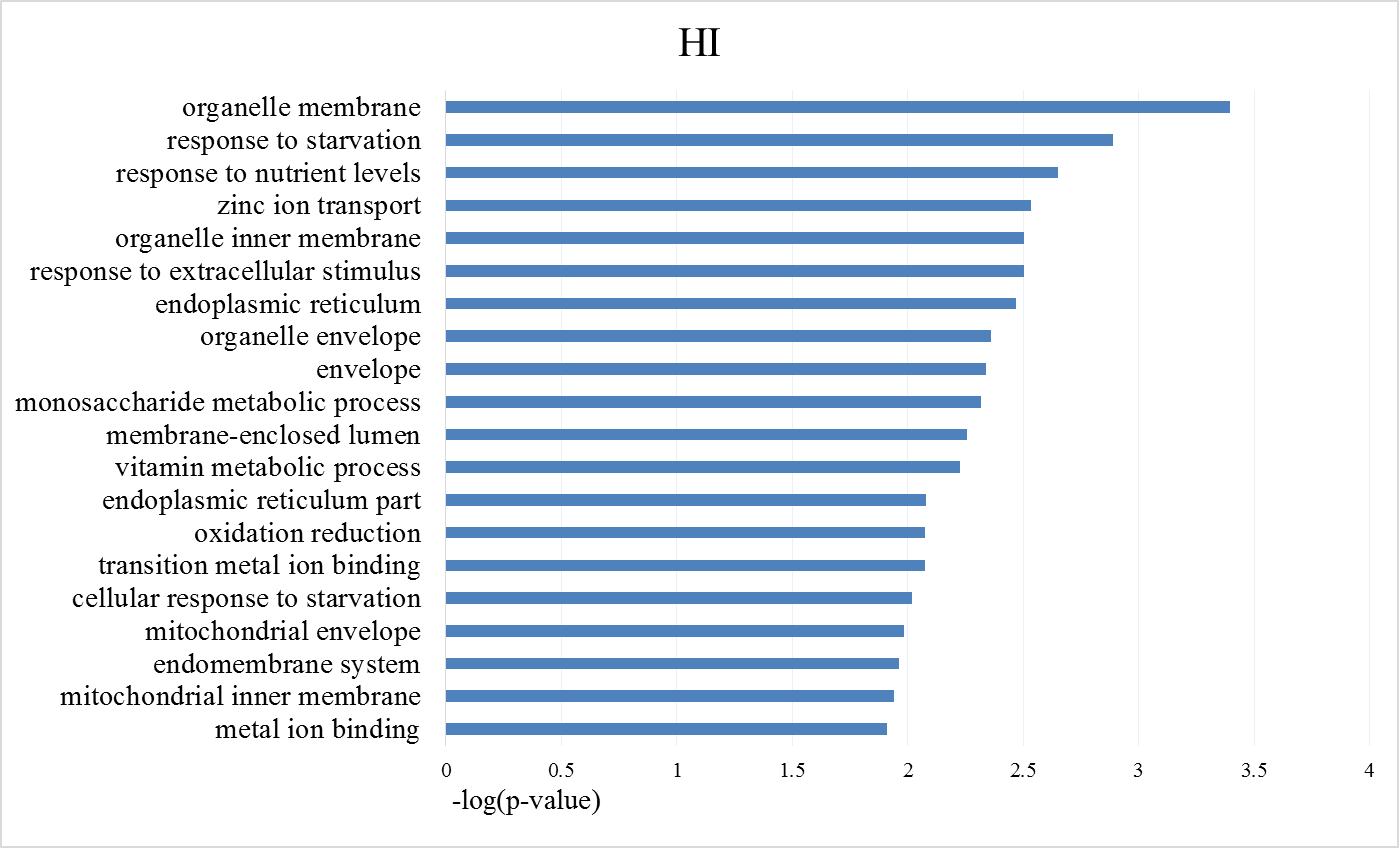
**

**Figure S7.** Gene Ontology enrichment analysis of DN-miRNA-mediated genes in high-glucose concentration.

**
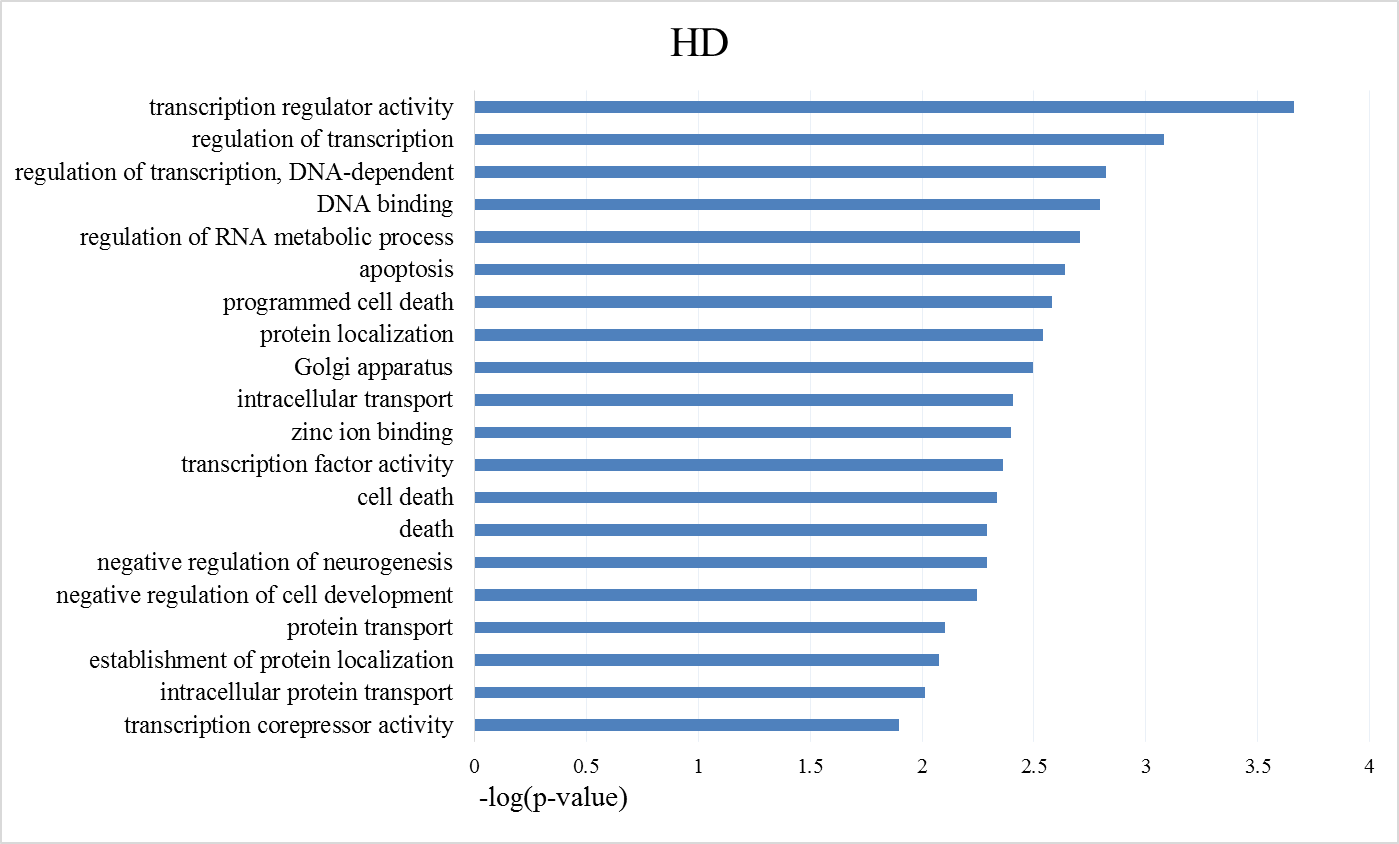
**

**Figure S8.** Gene Ontology enrichment analysis of UP-miRNA-mediated genes in high-glucose concentration.

**
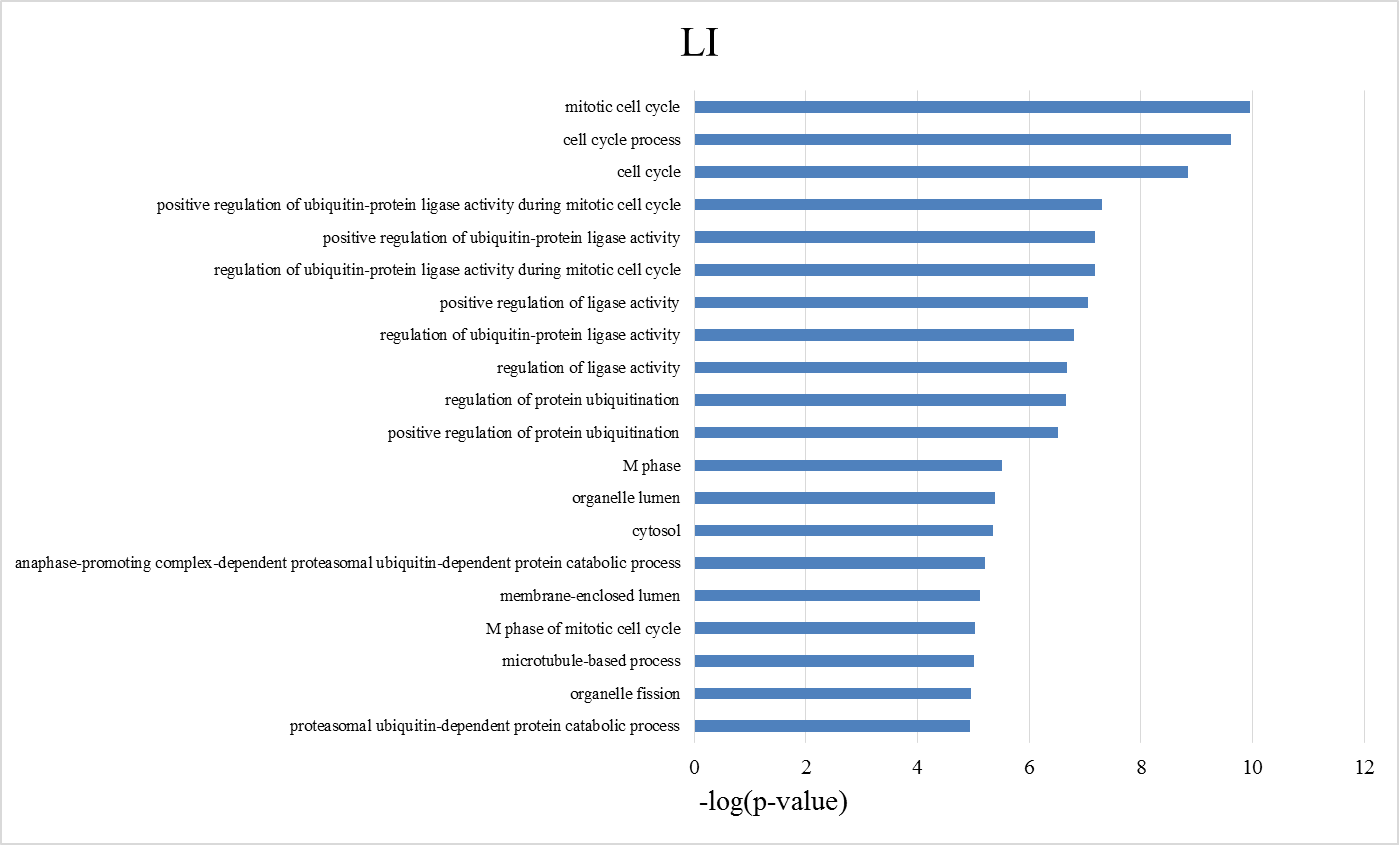
**

**Figure S9.** Gene Ontology enrichment analysis of DN-miRNA-mediated genes in low-glucose concentration.

**
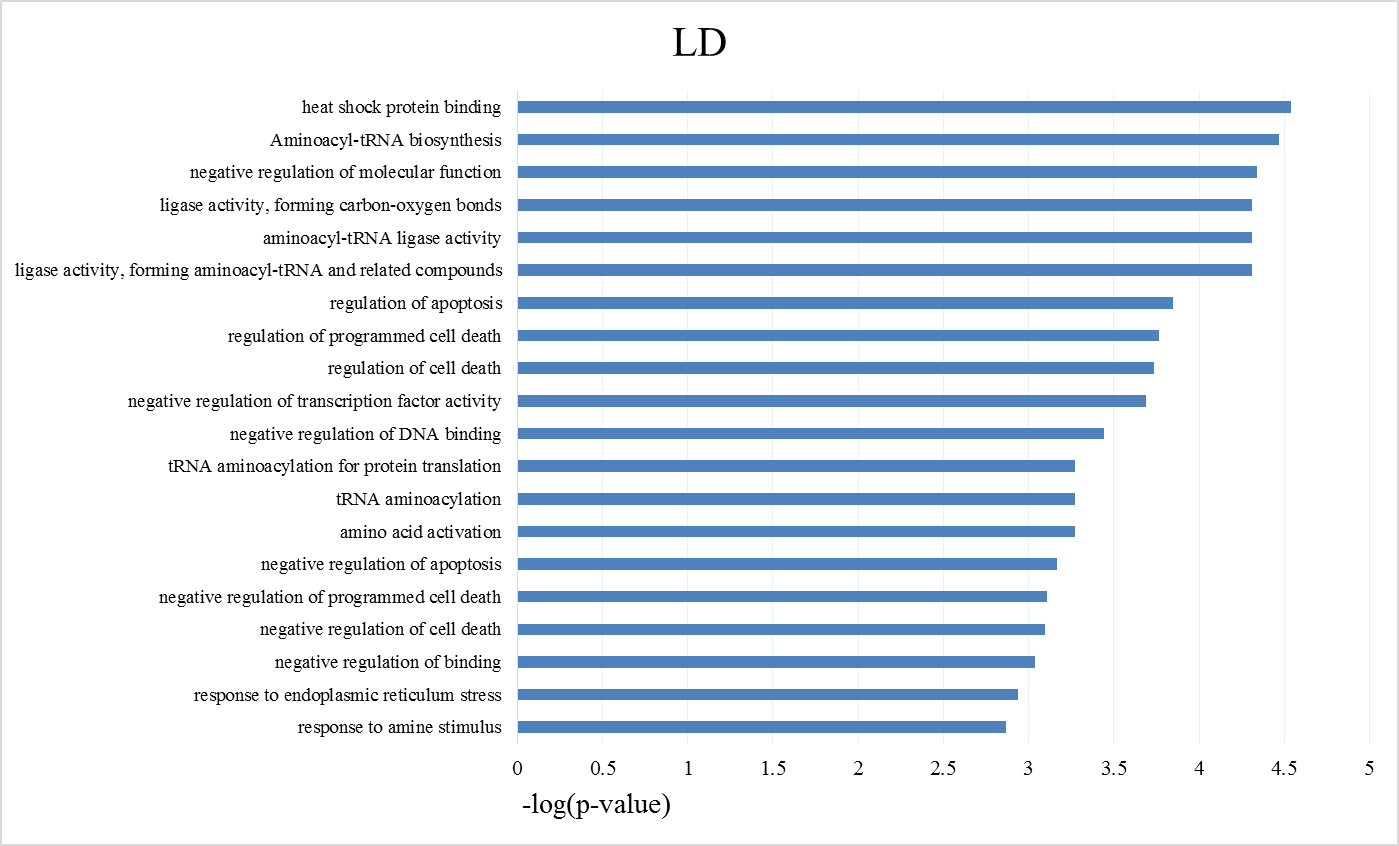
**

**Figure S10.** Gene Ontology enrichment analysis of UP-miRNA-mediated genes in low-glucose concentration.
